# Loss of function of folylpolyglutamate synthetase 1 reduces lignin content and improves cell wall digestibility in Arabidopsis

**DOI:** 10.1186/s13068-015-0403-z

**Published:** 2015-12-21

**Authors:** Avinash C. Srivastava, Fang Chen, Tui Ray, Sivakumar Pattathil, Maria J. Peña, Utku Avci, Hongjia Li, David V. Huhman, Jason Backe, Breeanna Urbanowicz, Jeffrey S. Miller, Mohamed Bedair, Charles E. Wyman, Lloyd W. Sumner, William S. York, Michael G. Hahn, Richard A. Dixon, Elison B. Blancaflor, Yuhong Tang

**Affiliations:** Plant Biology Division, The Samuel Roberts Noble Foundation, Ardmore, OK 73401 USA; BioEnergy Science Center, United States Department of Energy, Oak Ridge, TN 37831 USA; Complex Carbohydrate Research Center, University of Georgia, 315 Riverbend Road, Athens, GA 30602 USA; Department of Plant Biology, University of Georgia, Athens, GA 30602 USA; Center for Environmental Research and Technology (CE-CERT), Bourns College of Engineering, University of California, Riverside, CA 92507 USA; Department of Biological Sciences, University of North Texas, Denton, TX 76203 USA

**Keywords:** Arabidopsis, Bioenergy, C1 metabolism, Cell-wall recalcitrance, *FPGS1*, Lignin, Folylpolyglutamate synthetase

## Abstract

**Background:**

One-carbon (C1) metabolism is important for synthesizing a range of biologically important compounds that are essential for life. In plants, the C1 pathway is crucial for the synthesis of a large number of secondary metabolites, including lignin. Tetrahydrofolate and its derivatives, collectively referred to as folates, are crucial co-factors for C1 metabolic pathway enzymes. Given the link between the C1 and phenylpropanoid pathways, we evaluated whether folylpolyglutamate synthetase (FPGS), an enzyme that catalyzes the addition of a glutamate tail to folates to form folylpolyglutamates, can be a viable target for reducing cell wall recalcitrance in plants.

**Results:**

Consistent with its role in lignocellulosic formation, *FPGS1* was preferentially expressed in vascular tissues. Total lignin was low in *fpgs1* plants leading to higher saccharification efficiency of the mutant. The decrease in total lignin in *fpgs1* was mainly due to lower guaiacyl (G) lignin levels. Glycome profiling revealed subtle alterations in the cell walls of *fpgs1*. Further analyses of hemicellulosic polysaccharides by NMR showed that the degree of methylation of 4-*O*-methyl glucuronoxylan was reduced in the *fpgs1* mutant. Microarray analysis and real-time qRT-PCR revealed that transcripts of a number of genes in the C1 and lignin pathways had altered expression in *fpgs1* mutants. Consistent with the transcript changes of C1-related genes, a significant reduction in *S*-adenosyl-l-methionine content was detected in the *fpgs1* mutant. The modified expression of the various methyltransferases and lignin-related genes indicate possible feedback regulation of C1 pathway-mediated lignin biosynthesis.

**Conclusions:**

Our observations provide genetic and biochemical support for the importance of folylpolyglutamates in the lignocellulosic pathway and reinforces previous observations that targeting a single FPGS isoform for down-regulation leads to reduced lignin in plants. Because *fpgs1* mutants had no dramatic defects in above ground biomass, selective down-regulation of individual components of C1 metabolism is an approach that should be explored further for the improvement of lignocellulosic feedstocks.

**Electronic supplementary material:**

The online version of this article (doi:10.1186/s13068-015-0403-z) contains supplementary material, which is available to authorized users.

## Background

One-carbon (C1) metabolism is important for synthesizing a range of biologically important compounds such as purines and amino acids [1]. C1 enzymes use tetrahydrofolate and its derivatives, collectively referred to as folates, as cofactors for many of the biochemical reactions that they catalyze [2]. Methionine is an important product of the C1 metabolic pathway because it is the direct precursor of *S*-adenosyl l-methionine (AdoMet), the universal methyl-group donor, which is crucial for the synthesis of a large number of secondary metabolites [3], such as lignin, betaines, phytohormones [4], and hemicellulosic 4-*O*-methyl glucuronoxylan (GX) [5].

Lignin is a complex, polymeric phenylpropanoid-derived compound, which confers mechanical strength to the cell wall by cross-linking different polysaccharides, especially in fibers and tracheary elements [6]. The presence of lignin in secondary cell walls creates a hydrophobic environment in the vascular tissue for water conductance [7]. In plants, there are three major types of lignin monomers, namely p-coumaryl, coniferyl, and sinapyl alcohol [8]. Lignin biosynthesis is one of the most thoroughly investigated metabolic pathways in plants, but details on intermediate steps remain contentious and we have just started learning about the metabolic complexes that regulate flux through the phenylpropanoid pathway [9–12]. Recently, interaction of lignin with other wall matrix components has become a focus of cell wall research [13].

Lignin accounts for about 30 % of organic carbon on Earth [14] and is one of the major secondary compounds that utilizes a significant amount of AdoMet, the universal C1 pathway methyl donor. This has been shown in birch wood when the combined demands of several primary and secondary metabolites have been compared [3]. However, there is very little molecular evidence to show that these two pathways are directly linked. Although early radio-tracer experiments demonstrated that the methyl carbon of methionine, a precursor of AdoMet, was found in the *O*-methyl group of lignin [15], most of the studies to date have only provided indirect evidence linking C1 metabolism to lignin biosynthesis. For example, enriched transcripts of C1 metabolism genes in the lignin-rich vascular-tissues of various plant species were used to argue for a tight link between lignin biosynthesis and C1 reactions [16–18]. Only recently, mutant studies in model and crop plants are beginning to shed more light on the relationship between lignin biosynthesis and C1 metabolism. For instance, it was shown that a single-point mutation in the *S*-*adenosylmethionine synthetase3* (*SAMS3*) gene resulted in decreased AdoMet and lignin content in Arabidopsis [19]. More recent research in maize has demonstrated that a mutation in the *methylenetetrahydrofolate reductase* (*MTHFR*) gene resulted in reduced lignin [20].

The supply of methyl units is also important for the synthesis of other major components of secondary walls such as the hemicellulose 4-*O*-methyl glucuronoxylan (GX) [21]. A study conducted on Arabidopsis glucuronoxylan-specific methyltransferase (AtGXMT1- an enzyme that catalyzes 4-*O*-methylation of the glucuronic acid substituents) showed that AdoMet is the donor of methyl units to AtGXMT1 [5]. Therefore, C1 metabolism is vital for maintaining the proper synthesis of various polymers that are important in modulating the structure and physical properties of the cell wall.

In plants, the synthesis of folates involves three cellular compartments, the cytosol, mitochondria, and plastids [22]. Polyglutamylation of folates is essential for the cellular retention of this family of compounds and results in more efficient reactions for many folate-dependent enzymes [23, 24]. Most cellular folates carry a short poly-gamma-glutamate tail. Research has shown that removing these tails can reduce the total folate content up to 40 % in Arabidopsis [25] and cause major metabolic shifts and phenotypic changes in plants [26]. Folate polyglutamylation is carried out by the enzyme folylpolyglutamate synthetase (FPGS). Initial gene cloning, functional characterization, and cellular localization of the three isoforms of FPGS [*FPGS1* (plastid), *FPGS2* (mitochondria), and *FPGS3* (cytosol)] in Arabidopsis were described by Ravanel et al. [27]. Recently, the roles of *FPGS* genes in Arabidopsis have been explored through mutant analysis [24, 26, 28–31]. In addition to its impacts on early seedling development and root growth [24, 26, 30, 31], mutation of *FPGS1* caused changes in DNA methylation and the histone H3K9 dimethylation status of the Arabidopsis genome [29]. There is also a recent study showing that the maize *brown midrib 4* (*bm4*) mutant, which is disrupted in a gene encoding a functional FPGS, has reduced lignin. The lower lignin content in the *bm4* mutant further reinforces the importance of C1 pathway in lignin biosynthesis [32].

Previously, we showed that *fpgs1* mutants in *Arabidopsis* led to reduced levels of methionine and other C1 metabolic intermediates in young seedlings [26]. As a result, primary roots of the seedlings failed to develop properly. Despite the early root developmental defects, *fpgs1* mutants had above-ground growth comparable to wild-type plants [26]. Although there are recent reports that folate mutants in maize have reduced lignin [20, 32], it is not clear whether lower lignin resulting from altered folate metabolism leads to a corresponding reduction in cell-wall recalcitrance. Here we show that loss of FPGS1 function in Arabidopsis leads to lower lignin and reduced cell-wall recalcitrance. The reduced lignin observed in *fpgs1* mutants might not only be due to reduced flux of methyl units to lignin precursors, but is also a consequence of changes in the expression of genes associated with lignin biosynthesis and cell wall remodeling. These changes, in turn, result in plants with enhanced digestibility and sugar release efficiency, which are important requirements for efficient biofuel processing.

## Results

### *FPGS1* is preferentially expressed in vascular tissues, consistent with its role in lignin biosynthesis

**Fig. 1:**
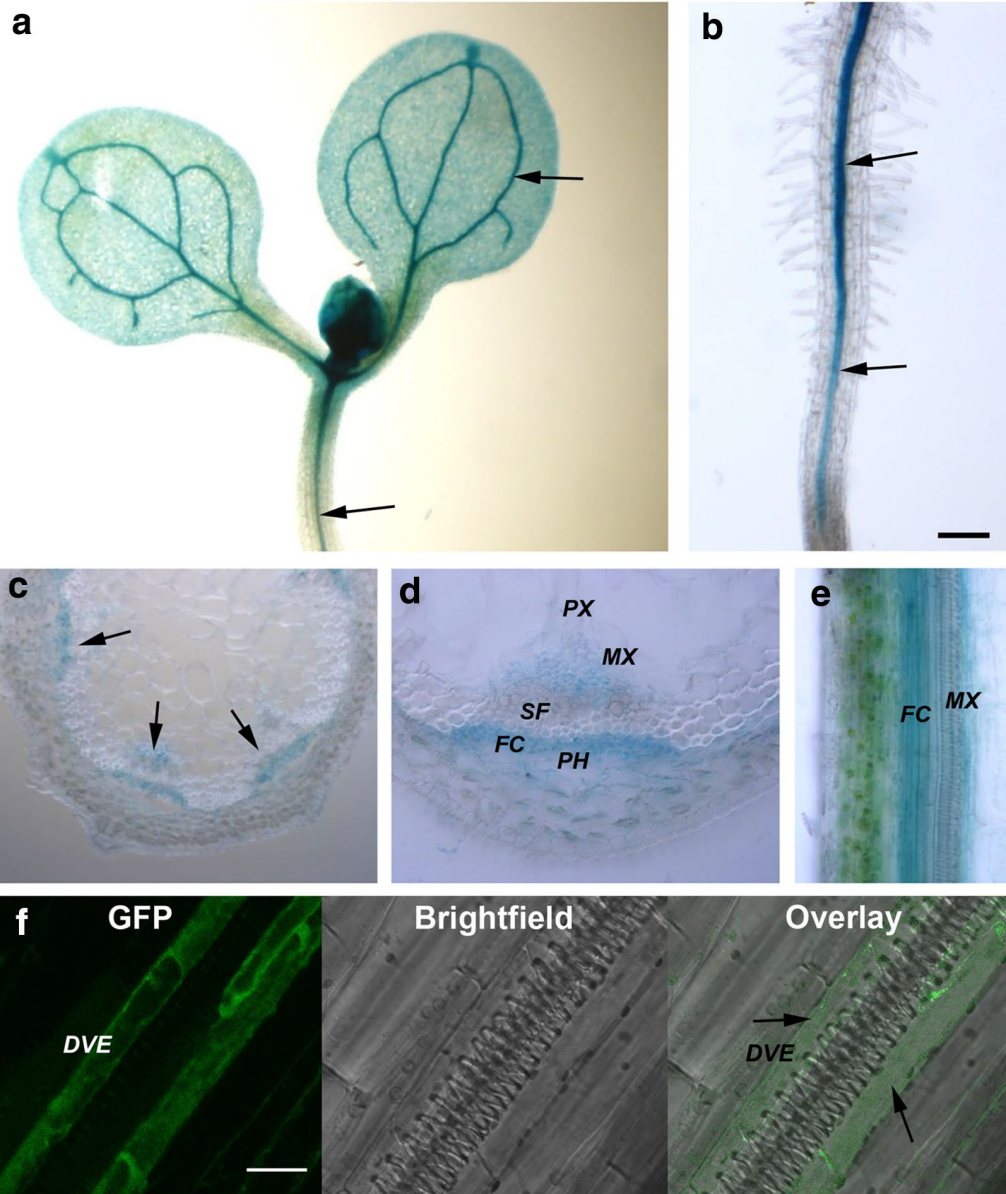
Expression pattern of *AtFPGS1*. **a**, **b** Plants transformed with *pFPGS1::GUS* constructs showing *FPGS1* expression in the vascular bundles of cotyledons and hypocotyls (**a**) and roots (**b**) of young seedlings. **c**, **d** Cross sections of the stained transgenic inflorescence stems showing *FPGS1* expression in the fascicular cambium and xylem tissue between protoxylem and metaxylem. **e** Longitudinal section of *pFPGS1::GUS* transgenic plants showing *FPGS1* expression in the fascicular cambium and xylem region adjacent to metaxylem. **f** Longitudinal inflorescence stem-sections (100 µm) of plants expressing *pFPGS1::FPGS1*-*GFP* were examined for GFP fluorescence. GFP signals were mainly detected in the developing vessel elements adjacent to the differentiated metaxylem. Superimposed image of GFP over a light microscopy image shows the locations of metaxylem and the GFP expressing cells. *Arrows* highlight the vascular tissue where *FPGS1* expression signal is detected in the cytosol. Phloem (PH), protoxylem (PX), metaxylem (MX), developing vessel element (DVE), sclerenchyma fiber (SF), and fascicular cambium (FC). *Scale bar* 20 µm

Lignification in plants occurs predominantly in the vascular tissues where secondary cell walls are formed. It has been shown previously that several C1 pathway genes that supply methyl units for lignin biosynthesis were enriched in the vascular tissues [17]. Consistent with previous reports, we found that the *FPGS1* promoter fused to *β*-*glucuronidase* (*GUS*) (*pFPGS1::GUS)* was predominantly expressed in the vascular tissues of cotyledons, hypocotyls, roots of seedlings and inflorescence stems (Fig. 1a–e). Based on both cross- and longitudinal sections of the transgenic plant inflorescence stems, GUS staining was mainly concentrated at the fascicular cambium region and the transition tissues from protoxylem to metaxylem (Fig. 1c–e).

The *FPGS1* expression pattern was further examined using green fluorescent protein (GFP). The entire sequence of *FPGS1* consisting of the 7-kb genomic DNA fragment was fused to GFP and transformed into the *fpgs1* mutant. The transgenic lines carrying *FPGS1 promoter::FPGS1*-*GFP* (*pFPGS1::FPGS1*-*GFP*) construct revealed that *FPGS1* expression was most distinct in the cytosol of developing vessel elements adjacent to the metaxylem (Fig. 1f). We previously showed that *pFPGS1::FPGS1*-*GFP* is localized in both chloroplasts and the cytosol when transiently expressed in tobacco leaf epidermal cells [33]. This observation is consistent with earlier reports that the Arabidopsis FPGS1 is located in plastids and the cytosol [30]. The *pFPGS1::FPGS1*-*GFP* was shown to complement the seedling root architectural defects of the *fpgs1* mutant; however, it localized mostly to the cytosol in roots [30] similar to observations made here in developing vessel elements. The observed *FPGS1* expression in the vascular tissues from GUS and GFP-reporter constructs was further supported by our earlier published in situ hybridization results [26] and publicly available microarray data sets [34] (Additional file 1: Fig. S1).

Unlike *FGPS1*, expression of the other two Arabidopsis FPGS isoforms (i.e. *FPGS*2 and *FPGS*3) is not particularly high in stems compared to other tissues. *FPGS2* is most highly expressed in leaves, whereas *FPGS3* is most predominantly expressed in rosette leaves and floral organs as suggested by publicly available microarray data sets [34]. Publicly available microarray data sets also show that *FPGS2* and *FPGS3* have lower expression in vascular tissues compared to *FPGS1* [34].

### Lignin content is reduced in *fpgs1* mutants

**Fig. 2:**
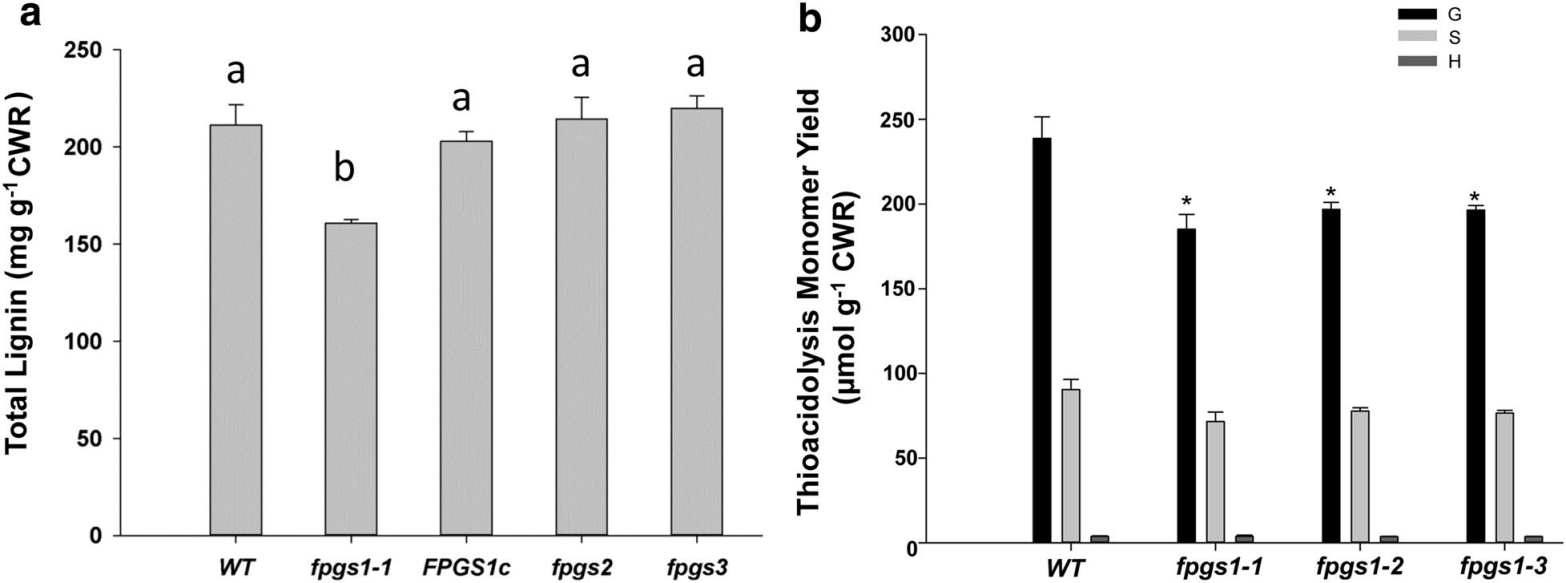
Lignin content and composition analysis in the 35-day-old stems of wild type, *fpgs1* and related lines. **a** Alterations in total lignin content measured by the acetyl bromide (AcBr) method in *fpgs1* lines, knockout mutants of the other two isoforms of *fpgs* (*fpgs2* and *fpgs3*), and complemented *FPGS1c*. Results showed that total lignin was significantly less in the *fpgs1* mutants and returned to the WT level in *FPGS1c*. Each data point was collected from five biological replicates. Each biological replicate had mature inflorescence stems of 20 individual plants (pooled). Means with different letters are statistically significant (Tukey’s test, *P* < 0.05). **b** Levels of S, G and H units were measured by the thioacidolysis method in all three mutant alleles of *fpgs1* (*fpgs1*-*1, fpgs1*-*2, fpgs1*-*3*) and compared with the wild-type plants. For each biological replicate, mature inflorescence stems of 20 individual plants were pooled and assayed for lignin monomers. The data represent the average value of five biological repeats for the mutants and wild type (WT) (±SE). *Statistically significant difference; *t* test (*P* < 0.05)

Metabolic profiling revealed that levels of many phenolic acids (cinnamic, p-coumaric, ferulic, and sinapic acids), as well as phenylalanine and coniferyl alcohol, were significantly reduced in 7-day-old *fpgs1*-*1* mutants compared to wild type ([26]; Additional file 2: Fig. S2). These data prompted us to examine lignin content and composition in the *fpgs1*-*1* mutants. To complement our lignin histochemical assays, lignin levels were quantified using the acetyl bromide (AcBr) method. We found a 17 % reduction in total lignin content in 35-day-old *fpgs1* plants compared to controls (Fig. 2). Lignin content of mutants of the other two isoforms of the three-membered *ArabidopsisFPGS* family (*fpgs2* and *fpgs3*) did not differ from that of the wild type (Fig. 2a). Two other *fpgs1* mutant alleles, namely *fpgs1*-*2* and *fpgs1*-*3*, also exhibited a significant reduction in total lignin (Fig. 2b). Additional evidence that a mutation in *FPGS1* was the cause of the lower lignin levels in mutant plants was obtained by introducing the *pFPGS1::FPGS1*-*GFP* construct into the *fpgs1*-*1* background. The *fpgs1*-*1* plants expressing the *pFPGS1::FPGS1*-*GFP* construct restored lignin to wild-type levels (Fig. 2a). The thioacidolysis method was used to determine lignin composition in the *fpgs1* mutants and wild type. Compared with the wild-type plants, a 22.5, 17.7, and 17.8 % reduction in G-lignin monomer was observed in the three *fpgs1* mutant alleles accordingly. No significant changes in S- or H-lignin monomers were observed in all *fpgs1* mutant alleles compared to the wild-type plants (Fig. 2b).

### Transcript profiling of *fpgs1* inflorescence stems reveals down-regulation of genes in the phenylpropanoid, methylation, and C1 pathways

To determine if changes in gene expression due to the *FPGS1* mutation could partly explain the reduced lignin phenotype of the *fpgs1* mutant, we conducted microarray analysis of 35-day-old inflorescence stem tissues using the ATH1 Affymetrix chip. Using a Bonferroni-corrected *P* value of 2.19202E−06 and a ratio of two as the cutoffs, there were 409 genes differentially expressed between the *fpgs1*-*1* mutants and wild-type plants. In the mutant, 182 genes had higher expression and 227 had lower expression (Additional file 3: Table S1/Sheet 2). The most up-regulated gene in the mutant was a member of the *R2R3*-*MYB* transcription factor gene family. Although the specific function of this *R2R3* gene is not known, several members of this family of transcription factors have been shown to regulate lignin biosynthesis [9, 10]. Two genes encoding *O*-methyltransferases and two encoding glycine-rich family proteins were among the top 10 most down-regulated genes in the mutant. At a ratio cutoff of two, 11 putative phenylpropanoid pathway-related genes were among the differentially expressed genes, and the majority of these were down-regulated, including one putative *caffeoyl*-*CoA 3*-*O*-*methyltransferase* (*CCoAOMT/*At1g67980) (Additional file 3: Table S1/Sheet 3).

A total of 16,484 probe sets were detected in the stem tissues based on the “Presence” call in at least four out of the six stem samples or in all three replicates in either wild-type or the *fpgs1* mutant based on the dCHIP program (http://www.hsph.harvard.edu/cli/complab/dchip/). Among them, 135 probe sets were known or homologous to known lignin pathway enzymes, based on the strategy of Costa et al. [35] (see http://cahnrs-cms.wsu.edu/ibc/research/lewis/nsf/Pages/Genes.aspx) (Additional file 3: Table S1/Sheet 2/sheet3). Using a ratio cutoff of 1.5, the expression of 25 probe sets annotated as known or putative lignin pathway genes (18.5 %, 2.53 times enrichment over the background) were affected in the *fpgs1* mutant. Similarly, 244 probe sets on the chip annotated as methyltransferases were detected in the stem tissue. Among the genes annotated as methyltransferases, the expression of 25 probe sets (10.3 %, 1.42 times enrichment over the background) was affected in the *fpgs1* mutant with most of them down-regulated (Additional file 3: Table S1/Sheet 4).

Among the genes directly involved in the C1 metabolic pathway, expression of *homocysteine S*-*methyltransferase3* (*HMT3*), which encodes an enzyme that converts homocysteine to methionine in the S-methylmethionine (SMM) cycle, was six times higher in the mutant. Similarly, the expression of *S*-*adenosylmethionine synthetase 3 and 4 (SAMS3, SAMS4)* was also up-regulated. On the other hand, expression of *SAMS1*, *SAMS2,* and several *AdoMet-dependent methyl transferases* was significantly down-regulated (Table 1; Additional file 3: Table S1). We also observed that significant numbers of genes involved in the sulfur assimilation pathway were affected in the mutant. Among 28 genes involved in sulfur metabolism, 13 were affected in the *fpgs1* mutant, with the majority of them up-regulated (Additional file 3: Table S1/Sheet 5).

**Table 1:** Relative expression levels of selected genes in wild-type and *fpgs1*-*1* plants estimated by two methods

Gene ID	Gene description	Real time qRT-PCR			Microarray
		Relative to internal standard		Ratio (*fpgs1*/WT)	Ratio (*fpgs1*/WT)
		*fpgs1*-*1*	WT		
AT1G67980	*S*-adenosyl-l-methionine: trans-caffeoyl Coenzyme A 3-*O*-methyltransferase	0.164	0.936	0.18*	0.281**
AT1G21100	Indole glucosinolate *O*-methyltransferase 1	0.665	2.411	0.28*	0.193**
AT3G54150	SAM-dependent methyltransferases superfamily protein	0.184	0.634	0.29*	0.270**
AT1G15125	SAM-dependent methyltransferases superfamily protein	2.591	8.325	0.31*	0.380**
AT1G69526	SAM-dependent methyltransferases superfamily protein	0.02	0.048	0.41*	0.500**
AT1G33030	Caffeic acid 3-*O*-methyltransferase family protein	0.126	0.288	0.44*	0.449**
AT4G22530	SAM-dependent methyltransferases superfamily protein	0.08	0.174	0.46*	0.491**
AT2G38080	Laccase 4	23.317	33.473	0.70*	0.97
AT1G02500	*S*-Adenosylmethionine synthetase 1 (*SAMS1*)	180.713	255.93	0.71*	0.96
AT1G66690	SAM-dependent methyltransferases superfamily protein	0.004	0.005	0.71*	0.65**
AT4G01850	*S*-Adenosylmethionine synthetase 2 (*SAMS2*)	10.468	14.607	0.72*	0.85**
AT1G15950	Cinnamoyl CoA reductase 1	23.261	31.304	0.74*	1.12
AT5G04230	*l*-Phenylalanine ammonia-lyase 3	0.275	0.354	0.78*	0.67**
AT1G72680	Cinnamyl alcohol dehydrogenase 1	0.558	0.693	0.80*	0.89
AT2G36880	Methionine sdenosyltransferase 3 (*SAMS4*)	9.275	6.237	1.49*	1.35**
AT3G17390	*S*-Adenosylmethionine synthetase 3 (*SAMS3*)	10.637	6.958	1.53*	1.32**
AT3G22740	Homocysteine *S*-methyltransferase 3 (*HMT3*)	0.303	0.035	8.70*	6.158**

* Student *t* test *P* < 0.05

** Associative analysis *P* < 2.20E−06 (Bonferroni corrected *P* value cutoff)

We validated our microarray data by quantitative real-time qRT-PCR in 35-day-old stem tissues of *fpgs1*-*1* and wild-type plants, focusing on the genes potentially involved in lignin biosynthesis and C1 pathways. Twenty-two genes, including one putative *CCoAOMT (*AT1G67980), one *O*-methyltransferase family protein *(*AT1G33030), three *SAM synthetases* (AT3G17390, AT1G02500, AT4G01850), several *AdoMet*-*dependent methyltransferases superfamily protein* (AT3G54150, AT1G15125, AT1G69526, AT4G22530, AT1G66690), and *Laccase 4 (*AT2G38080) were tested and validated (Table 1).

### Levels of the methyl group donor AdoMet and the degree of methylation of GX were reduced in *fpgs1*

**Fig. 3:**
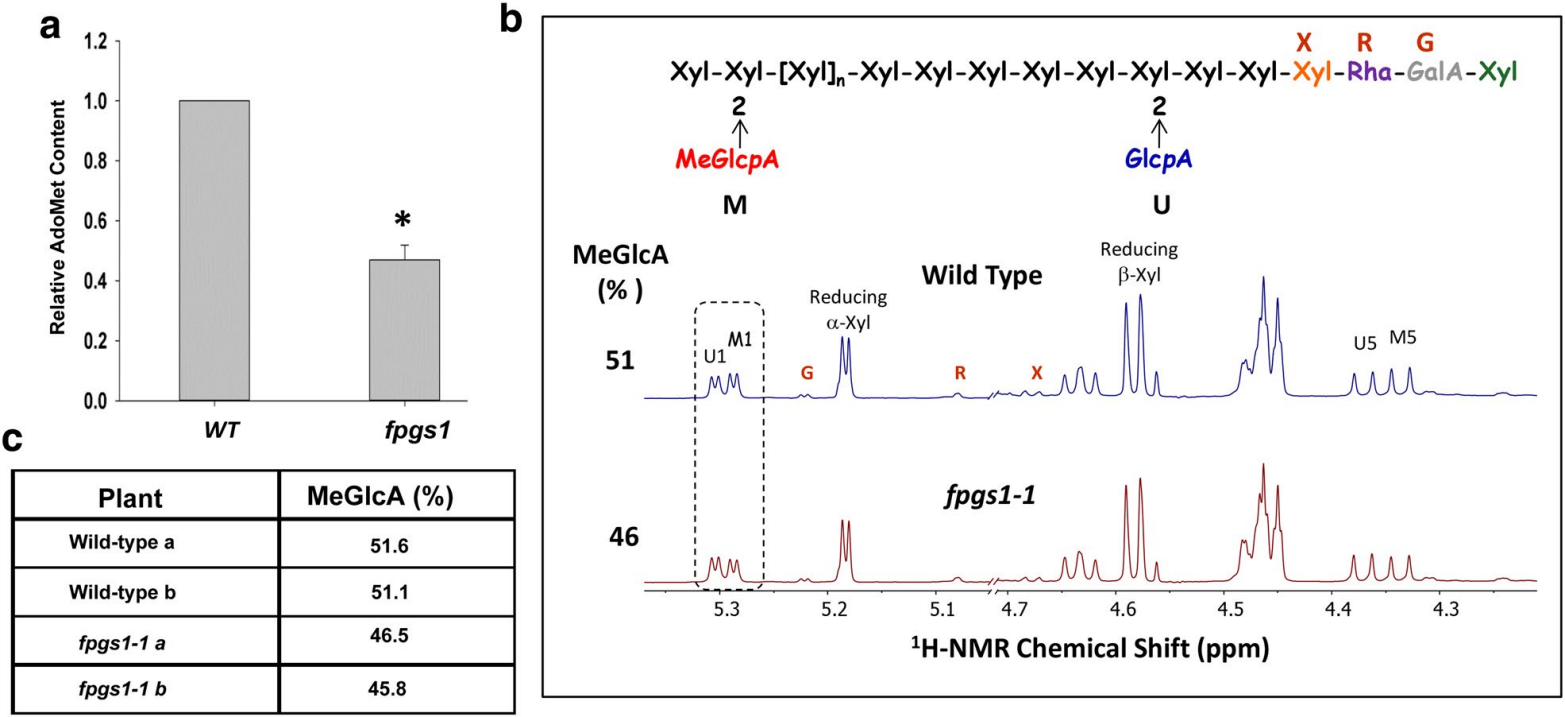
Relative S-adenosyl l-methionine (AdoMet) content and the degree of GX methylation in *fpgs1* and wild-type plants. **a** SAM content in wild-type (WT) and *fpgs1* stem tissue samples analyzed according to the method of Castro et al. [37]; a twofold reduction in AdoMet was observed in the *fpgs1*-*1* compared to WT plants. Five biological replicates were used during the measurement. **b** Determination of the degree of GlcA *O*-methylation of 4-*O*-methyl glucuronoxylan (GX) isolated from *fpgs1*-*1* and wild-type stem cell walls. Xylo-oligosaccharides were generated by endoxylanase treatment of the 1 N KOH-soluble GXs and analyzed using 600-MHz 1H NMR spectroscopy. The H-1 and H-5 resonances of α-D-GlcpA residues are labeled U1 and U5, respectively. The M1 and M5 labels correspond to H-1 and H-5 of 4-O-methyl α-D-GlcpA. Resonances assigned as H-1 of α-D-GalpA, α-L-Rhap, and β-D-Xylp linked to Rha residues are labeled G, R and X, respectively. The degree of GlcA methylation was determined by integration of U1 and M1 in the 1-D spectra (*dotted box*). **c** The NMR analyses of GX oligosaccharides were generated by two independent endoxylanase treatments showed a 10 % reduction of the degree of GlcA *O*-methylation of *fpgs1*-*1* GX compared with wild-type GX

AdoMet is the major methyl-group donor for numerous transmethylation reactions [36]. Methionine is the immediate precursor of AdoMet and we have shown previously that methionine levels are reduced in *fpgs1* mutant seedlings [26]. To determine if the mutation in *FPGS1* also affects AdoMet content, we quantified AdoMet by HPLC based on the method described by Castro et al. ([37]; Additional file 4: Fig. S3). Extracts from *fpgs1*-*1* mutant stems had about 50 % less AdoMet content than extracts from wild-type stems (Fig. 3a).

GX and lignin are the major polymers in the secondary cell wall that contain methyl groups. To determine if the reduced AdoMet content found in the *fpgs1*-*1* stems alters xylan methyletherification, we analyzed the structure of GX isolated from 35-day-old inflorescence stems of wild-type and *fpgs1*-*1* mutant by ^1^H-NMR spectroscopy, as described previously [5]. The NMR analyses showed that the degree of GlcA *O*-methylation of GX in *fpgs1*-*1* was 10 % lower than wild type (Fig. 3b, c). Other GX structural features, including the amount of branching and degree of polymerization were identical.

### Glycome profiling reveals subtle alterations in the cell walls of *fpgs1* inflorescence stems

**Fig. 4:**
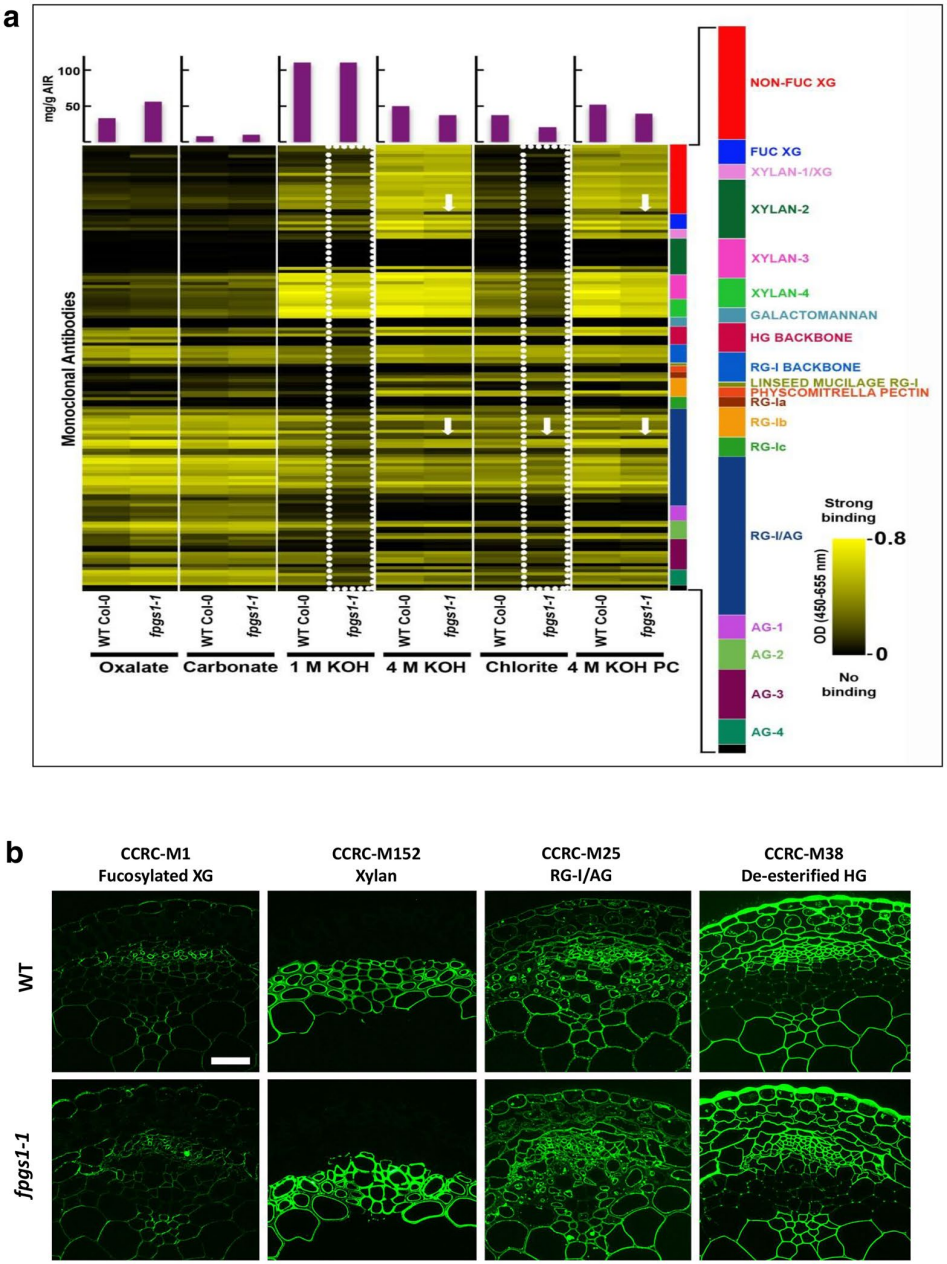
Immunological analyses of stems harvested from wild-type and *fpgs1*-*1* plants. **a** Glycome profiling of sequential extracts prepared from the cell walls isolated from 35-day-old stems of *fpgs1*-*1* and wild-type plants. The data are the average of three independent biological replicates. The various extraction reagents used are indicated at the *bottom* of the *figure*. The *bar graphs* above show amounts of carbohydrate materials released at each extraction step. Please note that these are sequential extracts (and not individual treatments) and reduced amounts released in last three extracts in mutant lines are potentially caused by the loss of excess carbohydrate materials in earlier lesser harsh extraction steps such as oxalate extraction. The panel on the *right* depicts the clades of monoclonal antibodies that recognize most major classes of plant cell wall glycans. The *dotted boxes* and *arrows* highlight the differences in the glycome profiles between *fpgs1*-*1* and wild-type plants. The *yellow-black scale* indicates the strength of the ELISA signal: *bright yellow color* depicts the strongest binding and the *black color* indicates no binding. **b** Immunofluorescence labeling with four selected antibodies representing different clades of glycans. Tissue was harvested from 35-day-old plants and fixed and sectioned as described in “Methods”. (*Scale bar* 25 µm and is applicable to all images)

Changes in lignin composition typically lead to corresponding modifications in other components of the cell wall [38–42]. To determine if there were any alterations in the overall extractability of non-cellulosic glycan epitopes in the *fpgs1*-*1* mutant, glycome profiling of the cell wall material (alcohol insoluble residues; AIR) isolated from 35-day-old inflorescence stems of wild-type and *fpgs1*-*1* mutants was conducted (Fig. 4a). The glycome profiles of the *fpgs1*-*1* mutants were mostly similar to those of wild-type lines. However, a subtle reduction in the overall abundance of xyloglucan (non-fucosylated and fucosylated) and xylan epitopes was observed in the 1 M KOH and chlorite extracts of *fpgs1*-*1* mutant walls (see white dotted blocks in Fig. 4a). Overall, when the total amounts of carbohydrate released during the various extractions were quantitated, it was noted that mild extractions released higher amounts of carbohydrate from cell walls of *fpgs1*-*1* inflorescence stems (significantly higher in oxalate and marginally higher in carbonate extracts) indicating that the extractability of cell walls from *fpgs1*-*1* was slightly higher than from wild type. Differences between *fpgs1*-*1* and wild-type wall extracts were also noted with respect to the binding of individual antibodies (see white arrows in Fig. 4a). Based on replicated data of glycome profiling, several mAbs that exhibited significant (student *t* test, *P* < 0.05) altered binding responses to the extracts from *fpgs1*-*1* mutants’ cell walls in comparison to wild type indicated changes in the abundances of their epitope structures in the extracts from mutants. For instance, in mutant cell wall extracts, enhanced binding responses were noted for some mAbs, including CCRC-M96 (belonging to the non-fucosylated xyloglucan-5 clade) in oxalate, chlorite, and PC 4 M KOH extracts; CCRC-M107 (belonging to the arabinogalactan-2 clade) in oxalate and carbonate extracts; and CCRC-M131 (belonging to the homogalacturonan backbone-1 clade) in carbonate extracts. Some mAbs exhibited reduced binding responses in mutant cell wall extracts, including CCRC-M90 (belonging to the non-fucosylated xyloglucan-6 clade) in 4 M KOH and PC 4 M KOH extracts; CCRC-M111 (belonging to the xylan-1/xyloglucan clade); CCRC-M24, CCRC-M25, and CCRC-M134 (all belonging to the RG-I/arabinogalactan clade) in the PC 4 M KOH extract (Additional file 5: Table S3). These data, in general, suggest potential cell wall alterations in *fpgs1*-*1*.

To determine if these minor differences in glycome profiling between wild type and *fpgs1*-*1* are reflected in situ, immunolabeling of cross-sections of 35-day-old inflorescence stems was conducted using monoclonal antibodies directed against a fucosylated xyloglucan epitope (CCRC-M1), a xylan epitope (CCRC-M152), an arabinogalactan side chain epitope of RG-I (CCRC-M25/RG-I/AG) and a non-methylesterified homogalacturonan epitope (CCRC-M38). All of these mAbs labeled wild-type and *fpgs1*-*1* mutant stems in a similar fashion, indicating no major compositional differences in the cell wall epitopes recognized by these antibodies between wild-type and the *fpgs1* mutants (Fig. 4b). It is worth noting that CCRC-M25 showed a significant difference in the glycome profiles for the PC 4 M KOH extracts, but such differences were not obvious in the in situ imaging.

Since homogalacturonan is another major sink for methyl groups, the difference in pectin methylation was examined by looking at the glycome profiling data for JIM5 and JIM7, antibodies that distinguish pectin with different degrees of methylation [43, 44], neither showed significance differences between wild type and *fpgs1*-*1*. This observation was consistent with immunolocalization of stem inflorescence sections, which also did not reveal differences in labeling intensities by these two antibodies between wild type and *fpgs1*-*1*. Taken together, our results suggest that subtle changes in wall extractability (as reflected in the glycome profiles) in the mutant are not accompanied by obvious changes in wall epitope composition, including pectin methylation.

### Saccharification efficiency is enhanced in *fpgs1* mutants

**Fig. 5:**
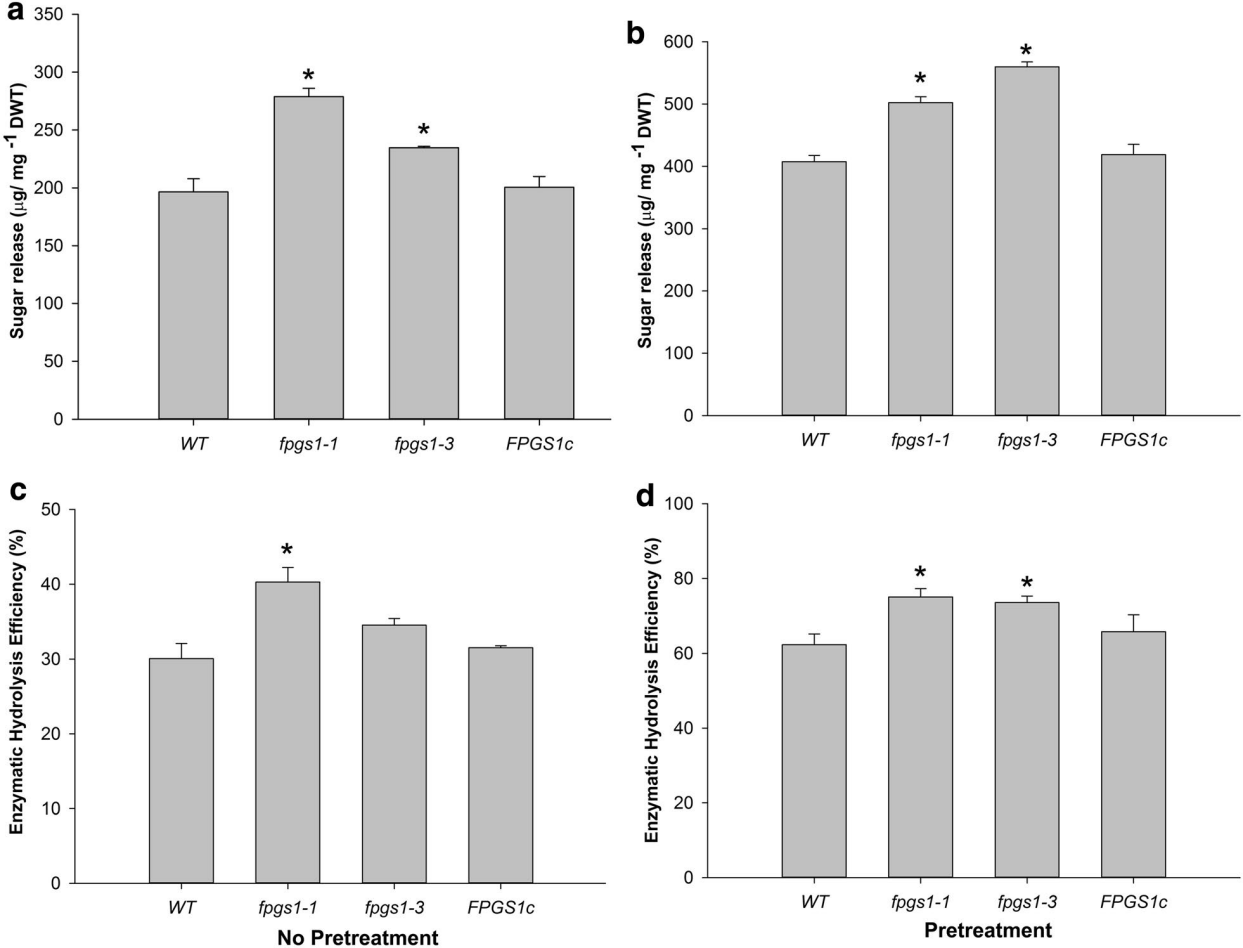
Glucose release and saccharification efficiency of *fpgs1* and wild-type plants. Total sugar release (**a**, **b**) and the saccharification efficiency (**c**, **d**) of *fpgs1*-*1*, *fpgs1*-*3*, wild type, and complemented *FPGS1c* plants without (**a**, **c**) or with (**b**, **d**) acid pretreatment, respectively. A total of 100 plants were used for this analysis. For each biological replicate, mature inflorescence stems of 20 individual plants were pooled and assayed for sugar release and the saccharification efficiency. The data represent the average value of five biological repeats for the mutants and wild type (WT) (±SE). Means with * are statistically significant over WT (Tukey’s test, *P* < 0.05)

Mature whole plants (stem and leaves) were analyzed for sugar release. Sugar release from the cell wall residues (CWRs) without acid pretreatment was 41 and 19 % more in the *fpgs1*-*1* and *fpgs1*-*3* plants, respectively, when compared to wild-type plants (Fig. 5a). Enzymatic hydrolysis of the CWR with acid pre-treatment showed 23 and 37 % higher sugar release in the *fpgs1*-*1* and *fpgs1*-*3* mutants, respectively, compared to wild-type plants (Fig. 5b). Accordingly, the saccharification efficiency of the mutant increased around 14–20 % without acid pre-treatment and around 18–34 % with acid pre-treatment (Fig. 5c, d). It is worth pointing out that the sugar release of the pFPGS1::FPGS1-GFP complemented lines (*FPGS1c*) was similar to the wild type, providing evidence that the improved saccharification efficiency in the *fpgs1* mutant is indeed directly attributable to the mutation.

An independent test was performed on wild-type and *fpgs1* CWRs using more sensitive HPLC methods. Although no major structural changes in the cell wall were noticed, a significant (5.88 %) increase in the glucan composition was observed in the *fpgs1*-*1* mutant (Additional file 6: Fig. S4A). Glucose and xylose released from hydrothermal pretreatment and co-hydrolysis were measured and we found a 24 and 8 % higher release of glucose and xylose, respectively, in the *fpgs1* mutant when samples were hydrothermally pretreated (Additional file 6: Fig. S4B), and 19 and 13 % higher release of glucose and xylose, respectively, when samples were digested without any pretreatment (Additional file 6: Fig. S4C).

## Discussion

Increasing cell wall digestibility has been achieved using various means [45, 46] but, mainly through the manipulation of genes encoding enzymes in the lignin biosynthetic pathway or those that directly modulate their expression [38–42]. Novel approaches are also being sought to reduce recalcitrance and methods such as partial substitution of normal monolignols with phenolic precursors or novel monolignols to increase carbohydrate accessibility, increase digestible sugar contents in the feedstock, and reduce substrate-derived fermentation inhibitors, all resulted in reduced recalcitrance [45, 46]. Here, we report that the *FPGS1* gene, a known component of C1 metabolism that supplies methyl units to lignin precursors and other cell wall components, can be an alternative target for reducing lignin content and cell wall recalcitrance. Although defects in growth were observed during early seedling development [26, 30, 31], such defects become less obvious in mature plants (Additional file 7: Fig. S5).

The *FPGS1* localization in the xylem suggested a possible involvement of *FPGS1* in lignin biosynthesis, as the xylem is a major site for this pathway. Other genes, such as *Acaulis5* [47], *SAMS3* [19]*, COMT,* and *CCoAOMT* [48–52], which are directly involved in xylem development or lignification, were also expressed in the vascular tissues. In wild-type Arabidopsis stem tissues, transcript levels of *FPGS1* were 27 times and 7 times more abundant than *FPGS2* and *FPGS3,* respectively. This indicates that *FPGS1* is the dominant FPGS isoform in the stem (Additional file 3: Table S1). The lower expression of *FPGS2* and *FPGS3* in stems and vascular tissues relative to *FPGS1* may explain why their corresponding mutants did not show reduced stem lignin. However, we cannot discount the possibility that other tissues in *fpgs2* and *fpgs3* mutants have reduced lignin.

In this study, we recorded approximately a fifty percent reduction of AdoMet in *fpgs1*-*1* stem tissues, which corresponded to about a 17 % reduction in lignin content. While a smaller impact on downstream product is generally expected due to concentrations, enzyme K_M_ and regulation, we cannot discount the possibility that there may be alternative sources of methyl units for the lignin biosynthetic pathway. In addition to those supplied by the other functional FPGS isoforms (FPGS2 and FPGS3), other sources of methyl units could be obtained through the *S*-methylmethionine (SMM) cycle. Through the tandem action of Met *S*-methyltransferase (MMT) and Hcy *S*-methyltransferase (HMT), the SMM cycle transports SMM from source tissues to sink tissues to be converted to methionine, which further replenishes the depleted AdoMet pools [3, 53, 54]. In the *fpgs1* mutant stems, transcripts of *HMT3* were up-regulated six times, indicating potential compensation of methyl units through the SMM cycle in the *fpgs1*.

The direct impact of the *FPGS1* gene product on AdoMet content could also be due to the fact that within the C1 pathway, methionine synthases cannot use monoglutamylated folates as methyl donors [55, 56]. Due to lower AdoMet levels in *fpgs1*, the conversion of cinnamic acid into monolignols via *O*-methylation reactions catalyzed by *CCoAOMT* or *COMTs* (Additional file 8: Fig. S6); [6, 57, 58] could be compromised. The reduced lignin content in *fpgs1* knockout plants mostly showed reduced levels of G-lignin and is reminiscent of previous reports where down-regulation of *CCoAOMT* resulted in less lignin because of the reduction mainly in G units in different species [6, 48–52, 59–63]. In the *fpgs1* mutant, transcript levels of *CCoAOMT1* (AT4G34050) or *COMT1* (AT5G54160) were not affected. It is worth noting, however, that transcripts of several *OMTs* and one putative *CCoAOMT* (AT1G67980) were significantly down-regulated in the *fpgs1* stem tissues (Additional file 3: Table S1). Although, direct involvement of these *OMTs* in the lignin biosynthesis has not been established, it is likely that some of these genes are involved in cell wall biosynthesis. Elucidating the function of these *OMT* genes will be the subject of future research.

Relationships among metabolic pathways have been shown in previous studies through transcriptomics analysis. For example, Loizeau et al. [64] found that antifolate methotrexate (MTX) treatment on Arabidopsis suspension cells induced significant changes in the expression of 4538 genes encoding FPGS, methionine synthases, methylene-THF reductases, *S*-adenosylmethionine synthetases, and methionine *S*-methyltransferases. Changes in the expression of these genes were associated with a significant modification in the distribution of C1 derivatives, resulting in the reorientation of C1 units towards the synthesis of critical biological compounds [64]. Recently, system-wide analysis of lignin biosynthesis pathway mutants also revealed that shikimate, phenylpropanoid, and methyl donor pathways are tightly co-regulated [41].

The essence of plastidial FPGS1 is not just restricted to the phenylpropanoid pathway and C1 metabolism, but also for the sulfur homeostasis in plastids. In support of this notion is the observation that changes in expression of nearly 13 out of the 28 genes involved in sulfur metabolism, including up-regulation of a few s*ulphate transporter* (*SULTR*) genes, occurred in the *fpgs1* mutants. Changes in the expression of *SULTR* genes are typically indicative of sulfur starvation [65]. It is noteworthy that methionine salvage and AdoMet play essential roles in sulfur, ethylene, and polyamine biosynthetic pathways [66]. The altered expression of genes involved in sulfur metabolism in *fpgs1* was not surprising because the plastid is the major organelle for sulfur metabolism and the two sulfur-containing amino acids, Met and cysteine, are key players in the C1 pathway. Sulfur deprivation in plastids could reduce the AdoMet content up to tenfold [67]. Changes in sulfur metabolism in the *fpgs1* mutant were also reflected by the fact that a significant number of genes involved in methionine-derived glucosinolate biosynthesis were up-regulated. Sulfate assimilation and glucosinolate biosynthetic are known to be interconnected [68]. Collectively, these metabolic shifts may have both direct and indirect impacts on the synthesis of lignin and secondary cell wall components.

In stem tissues, other pathways involved in secondary cell wall formation, such as the xylan biosynthetic pathway, also require methylation reactions [5, 69]. Our microarray analysis of the *fpgs1* mutant showed that several AdoMet-dependent methyltransferases, especially *O*-methyltransferases, were down-regulated in the mutant. The 10 % reduction in the GlcA *O*-methylation of GX in the *fpgs1*-*1* mutant further supports the involvement of *FPGS1* in secondary cell wall synthesis. The subtle changes in cell wall polysaccharide composition revealed by glycome profiling also indicate that changes in the C1 pathway may affect cell wall structure.

Reduction of lignin content or alteration of lignin composition has been shown to be associated with increased saccharification efficiency and reduced recalcitrance in many crops. For example, down-regulation of *COMT* and *CAD* increased saccharification efficiency in switchgrass by 16.5 and 23 %, respectively [70–72]. Furthermore, modification of various genes in the lignin biosynthesis pathway from different species has clearly demonstrated lignin down-regulation [38–42, 73–76]. Recent work in Arabidopsis using system biology approaches showed improved sugar release in most of the lignin down-regulated plants [42]. The partial redundancy in FPGS function could be exploited for the generation of biofuel crops with lowered lignin content and minimal undesired effects on biomass yield. It should be noted, however, that *FPGS2,* which encodes the mitochondrial FPGS isoform, is required for efficient nitrogen utilization [28], and recently, hypocotyls showed reduced growth in dark-grown *fpgs1* seedlings [31]. Thus, we cannot rule out the possibility that down-regulation of *FPGS1* could also lead to other plant developmental changes that are only manifested under certain environmental conditions. Nonetheless, the fact that *FPGS1* impacts lignin formation without any major adverse effects on plant growth makes it a promising target for improvement of lignocellulosic feedstocks for sustainable biofuel production.

## Conclusion

The present analysis of the Arabidopsis *fpgs1* mutants conclusively demonstrates that specific expression of FPGS1 in the vascular tissues contributes to the supply of methyl units for methylation reactions in the phenylpropanoid pathway. The increase in saccharification efficiency in the *fpgs1* mutant without dramatic negative effects on biomass traits points to an alternative target for improvement of lignocellulosic feedstocks. Our work also demonstrates that the essence of plastidial *FPGS1* is not just restricted to the lignocellulosic pathway, but also associated with the sulfur and glucosinolate pathways, which furthers our understanding of the complex regulation of plant secondary metabolism.

## Methods

### Characterization of *fpgs1* mutants and plasmid construction

The *fpgs1*-*1* (originally designated as *drh2 or atdfb*-*1*) mutant was identified through a forward genetic screen as described previously [26]. Two additional T-DNA insertion lines (SALK_015472, and SAIL_556_G08) at the *FPGS1* locus (*AT5G05980*) acquired from ABRC (https://abrc.osu.edu) were also used in this study and renamed as *fpgs1*-*2* and *fpgs1*-*3,* respectively. Plant growth and molecular characterization of all of these mutants were as described by Srivastava et al. [26]. Most of the analyses described here were conducted on the *fpgs1*-*1* mutant unless otherwise indicated.

All constructs were made using Gateway™ technology (Invitrogen, Now Life Technologies). The entry clones were obtained using the pENTR-D-TOPO vector (Invitrogen) and expression vectors using the Gateway system [77]. These were propagated in the *E. coli* strain *Top10* (Invitrogen) and DH5α, respectively. The templates used to clone all genes were genomic DNA or cDNA derived from *A. thaliana* Col-0 RNA. The 2-kb promoter region (upstream of the ATG start codon of *FPGS1*) was amplified using Taq DNA polymerase (Invitrogen), cloned into pENTR-D-TOPO, and later fused to uidA GUS reporter gene via LR-reaction in the destination vector pMDC162 for tissue expression analysis. The constructs were transformed into *Agrobacterium tumefaciens* strain LBA4404. For complementation studies, a 7-kb genomic DNA fragment containing the *pAtFPGS1*-*FPGS1* was amplified using gene-specific primers (Additional file 9: Table S2). This construct is comprised of 2 kb upstream promoter and 5 kb downstream regions of the ATG start codon. This was cloned into *pMDC107* binary vectors and transformed into *A. tumefaciens* LBA4404. The *fpgs1*-*1* mutant was then transformed using the floral-dip method [78]. Transgenic plants were selected on 25 μg ml^−1^ hygromycin plates and later propagated on soil. Around ten independent T2 transgenic lines were tested and analyzed for fluorescence and complementation.

### Lignin analysis

Lignin content and composition were measured in the post-flowering 35-day-old stem tissues by the AcBr [79] and thioacidolysis [80] methods. At this stage, plants had well-developed inflorescence stems, which was optimal for lignin quantification. Because 35-day-old plants were still mostly green, this time point was chosen so we could obtain quality RNA for gene expression data that corresponded to lignin analysis. Lignin-derived monomers were identified and quantified by GC/MS using a Hewlett–Packard 5890 series II gas chromatograph with a 5971 series mass selective detector (column: HP-1, 60 m × 0.25 mm × 0.25 μm film thickness). Mass spectra were recorded in electron impact mode (70 eV) with 60–650 *m*/*z* scanning range. Five biological replicates were included for each assay. For each biological replicate, mature inflorescence stems of 20 individual plants were pooled and assayed.

### Microscope image acquisition

Confocal imaging was performed using a Leica TCS SP2 confocal microscope (Leica Microsystems) and the HCX PL APO 63 ×/1.2 W water-immersion objective. All images were analyzed using Leica Confocal Software. GFP channels were acquired by scanning sections using the 488-nm laser line for excitation. Emission of the GFP signal was detected between 500 and 530 nm. Microscopy of vascular tissues was carried out after preparing 100 µm longitudinal sections of 35-day-old fresh stem tissues using Vibratome 1000 plus. GUS staining was performed on 35-day-old stem tissues and 7-day-old whole seedlings as described in Srivastava et al. [26].

### Affymetrix microarray and qRT-PCR analysis

The Affymetrix arrays and qRT-PCR experiments were performed using total RNA from 35-day-old inflorescence stem basal portion. Three biological replicates were included for both wild-type control and *fpgs1* mutant and each replicate had ten pooled plants. Total RNA was isolated using an RNeasy Mini Kit (Qiagen, Valencia, CA). RNA quantification and QC was as described previously [81]. For each array experiment, 500 ng of total RNA was used for labeling using the Affymetrix GeneChip^®^ Arabidopsis ATH1 Genome Array (Affymetrix, Santa Clara, CA). Probe labeling, chip hybridization, and scanning were performed according to the manufacturer’s instructions for IVT Express Labeling Kit (Affymetrix). Data normalization and analysis were conducted as described earlier [81]. The microarray data were submitted to ArrayExpress and the accession no. is E-MEXP-3915.

Real-time qRT-PCR was performed using previously described procedures [26]. The plant-specific EF4A2 (At1g54270) gene was used as a control for constitutive gene expression. The primers used for this analysis are presented in Additional file 9: Table S2. Three biological replicates and three technical replicates were used for each sample group.

### Analysis of AdoMet levels

Two hundred mg lyophilized ground samples of wild type and the mutant stem tissue were put into 10 ml ethanol: water (70:30 v/v) mix and samples were centrifuged at 14,000 rpm for 15 min at 4 °C. Supernatant was collected and further processing and analysis of the sample were done as described by Castro et al. [37]. UPLC/FLR analysis of AdoMet derivatives was carried out using a Waters Acquity UPLC system (Waters) coupled to FLR detector (Waters). A Waters Acquity UPLC 2.1 × 150 mm, BEH C18 column was used for analysis at 20 °C. The mobile phase consisted of 0.1 M sodium acetate in HPLC grade water, pH 4.5 (A), and HPLC grade acetonitrile (B). Using an injection volume of 2.0 µl, separation was achieved using the following gradient: isocratic at 95.8 % A for 7.8 min; 95.8 % A to 50 % A in 6 min; isocratic at 50 % A for 1 min followed by re-equilibration at 95.8 % A for 7 min at a flow rate of 0.280 ml/min. The fluorescent AdoMet derivatives were monitored at excitation of 270 nm and emission of 410 nm with a PMT Gain setting of 1.00 and data rate of 5 pts/s.

A total of five biological replicates were used, and 20 plants in total were pooled for each replicate. Identification of compounds was based on retention time of authentic standards. Data were processed using Empower 2 Software (Waters).

### Preparation of glucuronoxylan oligosaccharides and ^1^H-NMR spectroscopy

Alcohol insoluble residues (AIR) were obtained from 35-day-old inflorescence stems of wild-type and *fpgs1*-*1* mutant and treated sequentially with specific endoglycanases as described [82]. The enzyme-treated residue was then extracted with 1 N KOH containing 1 % (w/v) NaBH_4_ and then with 4 N KOH containing 1 % (w/v) NaBH_4_. The 1 and 4 N KOH soluble fractions were neutralized with glacial acetic acid, dialyzed against deionized water and lyophilized. Glucuronoxylan oligosaccharides were generated by treating the 1 N KOH soluble fractions for 24 h at 37 °C with a *Trichoderma viride* endoxylanase (M1, 0.04 units/10 mg polysaccharide; Megazyme).

The glucuronoxylan-derived oligosaccharides were characterized by ^1^H NMR spectroscopy as described in Urbanowicz et al. [5]. Glucuronoxylan oligosaccharides (1–2 mg) were dissolved in D_2_O (0.25 mL, 99.9 %; Cambridge Isotope Laboratories, Andover, MA). One- and two-dimensional NMR spectra were recorded at 298°K with a Varian Inova-NMR spectrometer (Agilent Technologies, Santa Clara CA) operating at 600 MHz for ^1^H and equipped with a 5-mm NMR cold probe as described [5]. Chemical shifts were measured relative to internal acetone (*δ* 2.225). Data were processed using MestReNova software (Universidad de Santiago de Compostela, Spain). The extent of GlcA methylation was determined by integration of the signals corresponding to the anomeric protons of GlcA and methylated GlcA in the 1D NMR spectra. The areas of the overlapping signals were determined using the deconvolution method in MestReNova. The degree of GlcA *O*-methylation was calculated using the data obtained in the NMR analysis of GX oligosaccharides obtained by two independent endoxylanase treatments.

### Glycome profiling and immunofluorescence labeling

Preparation of cell wall AIR, cell wall fractionation, and glycome profiling were carried out as described previously [83, 84]. Plant glycan-directed monoclonal antibodies [85]; Additional file 10: Appendix S1) were obtained as hybridoma cell culture supernatants from laboratory stocks at the Complex Carbohydrate Research Center [CCRC, JIM, and MAC series; available from CarboSource Services (http://www.carbosource.net)]. For immuno-labeling experiments, 35-day-old stem from both wild type and *fgps1*-*1* were fixed, sectioned, and immunolabeled as described by Avci et al. [86].

### Determination of saccharification efficiency

Cell-wall residues (CWRs) used in lignin analysis were also used for sugar analyses as described previously [70, 71] with slight modifications. Enzymatic saccharification of Arabidopsis samples (with and without acid pre-treatment) was performed following the analytical procedure of the National Renewable Energy Laboratory (LAP-009) (http://www.nrel.gov/biomass/analytical_procedures.html). To release total sugar in the medium, 100 mg CWR was incubated at 30 °C for 1 h in 1.5 ml 72 % (w/v) H_2_SO_4_ and later autoclaved at 121 °C for 30 min after adding 42.5 ml deionized water. The material was centrifuged at 4000 rpm for 30 min and the supernatant was collected in a new tube followed by diluting the entire extract to 50 ml with deionized water. For the saccharifications without pretreatment, 100 mg CWR was directly incubated in 10 ml digestion solution (8.8 ml 0.1 M, pH = 4.8 sodium citrate buffer + 0.2 ml 2 % NaN_3_ + 1.0 ml Enzyme Cocktail stock) at 50 °C for 72 h. The enzyme cocktail stock was prepared using 1.5 ml cellulase stock from *Trichoderma reesei* (Sigma-Aldrich, St. Louis, MO, USA) and 1.5 ml Novozyme 188 stock Cellobiase from *Aspergillus niger* (Sigma-Aldrich) in 45.5 ml sodium citrate buffer (pH = 4.8). For pretreated samples, 100 mg CWRs were pretreated with 2.5 ml of 1.5 % (w/v) H_2_SO_4_ and these samples were autoclaved at 121 °C for 40 min. This acid-pretreated material was washed three times with 10 ml water to obtain neutral pH and after each washing, the material was centrifuged at 4000 rpm for 20 min to retain any CWRs from the supernatant. The remaining biomass was lyophilized for 24 h and treated with enzyme cocktail as described earlier. Monomeric sugars (glucose and xylose) released in the samples were analyzed spectrophotometrically using the phenol–sulfuric acid assay method [87]. The concentration in the original sample was calculated with a standard curve based on known *d*-glucose concentrations (Sigma-Aldrich).

Saccharification efficiency was determined as the ratio of sugars released by enzymatic hydrolysis to the amount of sugars present in the cell wall material before enzymatic hydrolysis. For each biological replicate, mature inflorescence stems of 20 individual plants were pooled and assayed. Five biological replicates were included for each assay.

### High-throughput pretreatment and enzymatic hydrolysis (HTPH) and downscaled compositional analysis

The HTPH system at University of California, Riverside [88–90] was employed to determine whether the mutants exhibited altered sugar release compared to the WT control during pretreatment and enzymatic hydrolysis. Glucose and xylose release from hydrothermal pretreatment and co-hydrolysis were measured. First, hydrothermal pretreatment was conducted at 180 °C for 11.1 min. Then, the pretreated slurry was co-hydrolyzed 50 °C in 50 mM citrate buffer (pH 4.8) for 72 h. Accellerase^®^ 1500 cellulase (112.5 mg) and XY xylanase (37.5 mg) from Genencor were used to release glucan and xylan in the raw biomass. Glucans and xylans were determined using a downscaled compositional analysis method [88] that is nearly identical to conventional wet chemistry procedures [90], but uses 100 times less biomass. The entire process was performed in 1.5 ml high recovery glass vials (Agilent, Santa Clara, CA, USA) with 3 mg dry biomass, loaded into each vial by a Core Module Robotics Platform (Symyx Technologies, Sunnyvale, CA). A set of glucose and xylose sugar recovery standards (SRS) were run in parallel for correction of sugar degradation. Sugars were analyzed by Waters Alliance 2695 HPLC (Milford, MA, USA) equipped with an Aminex HPX-87H column (BioRad, Hercules, CA, USA) and a refractive index detector.

## Additional files

10.1186/s13068-015-0403-z Publicly available microarray data showing preferential expression of *FPGS1* in vascular tissues. Data from Genevestigator (https://genevestigator.com/gv/).
10.1186/s13068-015-0403-z Metabolite profiling in 7-day-old whole seedlings of wild type and *fpgs1*-*1*. Results showing significant reduction in levels of lignin precursors and related compounds in the *fpgs1*-*1* mutant. Re-production permitted by Plant Physiology [26]. Each biological replicate was visualized in a single column (average of n = 3). Alterations in mean phenolic acid levels of *fpgs1*-*1* compared with the wild type (Student’s t test, * P < 0.05).
10.1186/s13068-015-0403-z Microarray analysis table of *fpgs1*-*1* and wild-type plants in 35-d-old stem of Arabidopsis.
10.1186/s13068-015-0403-z Chromatographic profiles of wild-type and *fpgs1* stem tissue samples analyzed for AdoMet content. Analysis was according to Castro et al. [37].
10.1186/s13068-015-0403-z mAbs detecting significant differences between wild-type and *fpgs1* mutants stem serial extracts.
10.1186/s13068-015-0403-z Hydrothermal pretreatment releases more xylose and glucose from *fpgs1* biomass than from wild-type biomass. (A) Glucan and xylan contents of Arabidopsis wild-type and *fpgs1*-*1* stem biomass. (B) Total xylose (monomer plus oligomers) released by cellulase and xylanase (150 mg protein/g structural sugars in biomass) after hydrothermal pretreatment at 180 °C for 11.1 min. (C) Glucose and xylose released by cellulase and xylanase (150 mg protein/g structural sugars in biomass) without pretreatment. Error bars are SE, n = 4.
10.1186/s13068-015-0403-z Comparative aerial growth analysis of *fpgs* mutants, complemented *FPGS1* line (*FPGS1c*) and wild-type plant. The growth experiment included wild-type plant, two independent mutant lines of *fpgs1* (*fpgs1*-*1* and *fpgs1*-*3*), knockout mutant of other two isoforms of *FPGS* genes (*fpgs2*-mitochondiral and *fpgs3*-cytoplasmic) and complemented transgenic lines of *fpgs1*-*1* [*FPGS1c* (*pFPGS1::FPGS1*-*GFP*::*fpgs1*-*1*)]. Samples were collected at 35 days after germination. Collectively no visual differences were observed in the above-ground growth of these plants (A). No significant differences were observed in per plant fresh weight (B) and dry biomass (C).
10.1186/s13068-015-0403-z Schematic representation of C1 metabolism association with the monolignol biosynthetic pathway (Adapted from Humphreys and Chapple [91], Ravanel et al. [56]; van den Broeck et al. [16]. Arrows represent enzymatic reactions and abbreviations depicted next to arrows are the name of the enzyme that catalyzes the associated reaction. (PAL) l-Phenylalanine ammonia lyase; (C4H) Cinnamate 4-hydroxylase; (4-CL) 4-coumarate:coenzyme A ligase; (CCR) Cinnamoyl-CoA reductase; (HCT) Hydroxycinnamoyl CoA: shikimate hydroxycinnamoyltransferase; (C3′H) 4-Coumaroyl-shikimate 3′-hydroxylase; (CCoAOMT) Caffeoyl-CoA-3-O-methyltransferase; (CAD) Cinnamyl alcohol dehydrogenase; (Lac 4) Laccase 4; (F5H) Ferulate 5-hydroxylase; (COMT) Caffeic acid 3-O-methyltransferase; (FPGS1) Folypolyglutamate synthetase 1; (SHMT) Serine hydroxymethyltransferase; (5,10-Met-THF) 5,10-Methylene-tetrahydrofolate reductase; (Met-Syn) Methionine synthase; (SAM-Syn) S-Adenosyl methionine-Synthase; (SAM-MT) S-Adenosyl methionine dependent Methyl transferase; (SAM-HCT) S-adenosyl homocysteinase; (SAHH) S-Adenosyl-L-homocysteine hydrolase; (Cys) Cysteine; (Cys-Syn) Cystathionine synthase; (Cys-Lys) Cystayhioninelyase.
10.1186/s13068-015-0403-z Primer sequences used for genotyping, plasmid construction, and gene expression analysis by qRT-PCR.
10.1186/s13068-015-0403-z List of cell wall monoclonal antibodies.

## Abbreviations

AdoMet: *S*-adenosyl l-methionine
AIR: alcohol insoluble residues
AtGXMT1: Arabidopsis glucuronoxylan methyltransferase 1
C1: one-carbon
*CCoAOMT*: *Caffeoyl*-*CoA 3*-*O*-*methyltransferase*
CCRC: Complex Carbohydrate Research Center
CWR: cell wall residue
FPGS: folylpolyglutamate synthetase
GFP: green fluorescent protein
*GUS*: *β*-*glucuronidase*
GX: 4-*O*-methyl glucuronoxylan
HMT: Hcy *S*-methyltransferase
*SAMS*: *S*-*adenosylmethionine synthetase*
SULTR: sulphate transporter
