# Supplementary figures and images for: Loss of function of folylpolyglutamate synthetase 1 reduces lignin content and improves cell wall digestibility in Arabidopsis
Source: Biotechnol Biofuels. 2015 Dec 21;8:224. doi: 10.1186/s13068-015-0403-z (PMC4687376; doi:10.1186/s13068-015-0403-z)

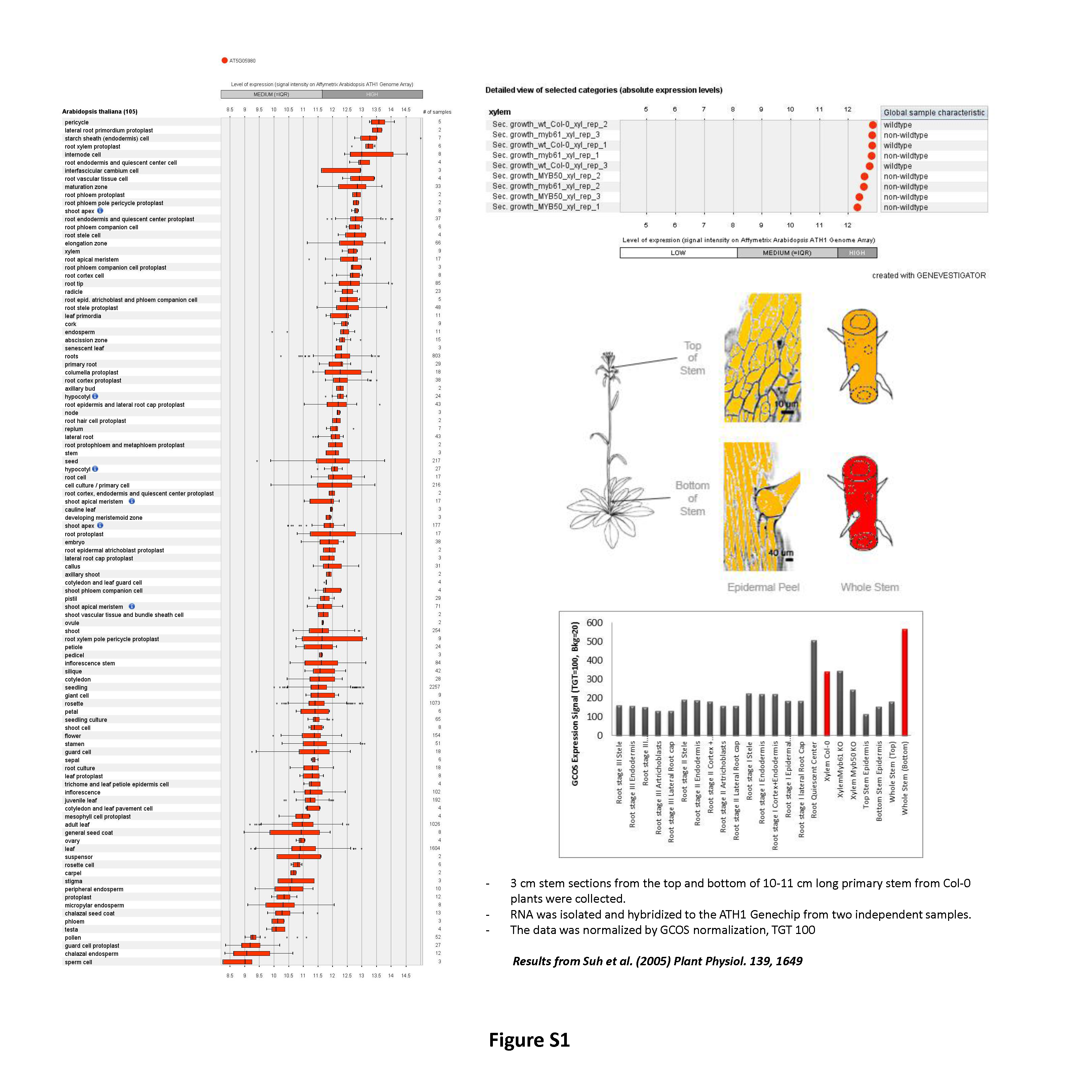

Supplement: Supplementary file 1 — 10.1186/s13068-015-0403-z Publicly available microarray data showing preferential expression of FPGS1 in vascular tissues. Data from Genevestigator (https://genevestigator.com/gv/). [file 13068_2015_403_MOESM1_ESM.tiff]

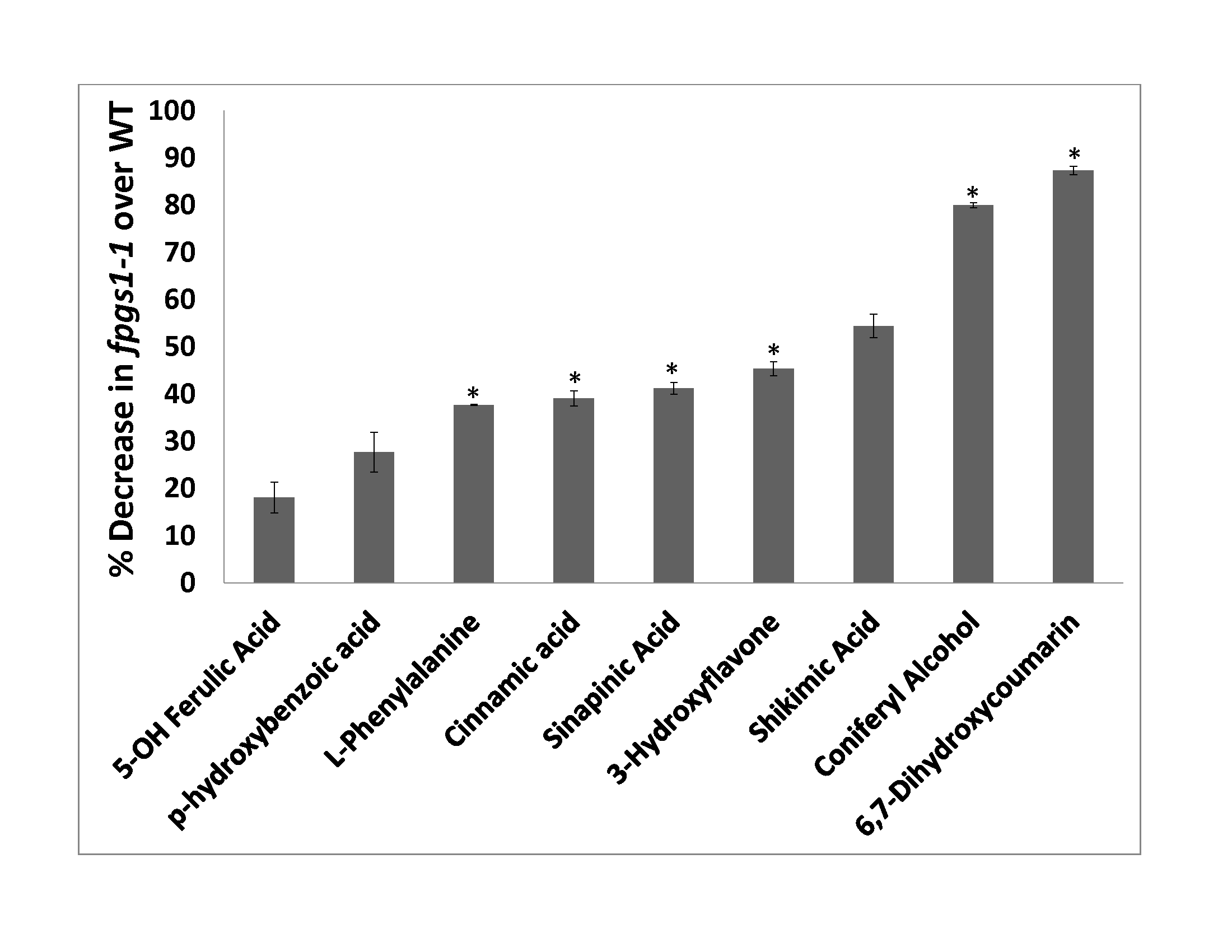

Supplement: Supplementary file 2 — 10.1186/s13068-015-0403-z Metabolite profiling in 7-day-old whole seedlings of wild type and fpgs1-1. Results showing significant reduction in levels of lignin precursors and related compounds in the fpgs1-1 mutant. Re-production permitted by Plant Physiology [26]. Each biological replicate was visualized in a single column (average of n = 3). Alterations in mean phenolic acid levels of fpgs1-1 compared with the wild type (Student’s t test, * P < 0.05). [file 13068_2015_403_MOESM2_ESM.tiff]

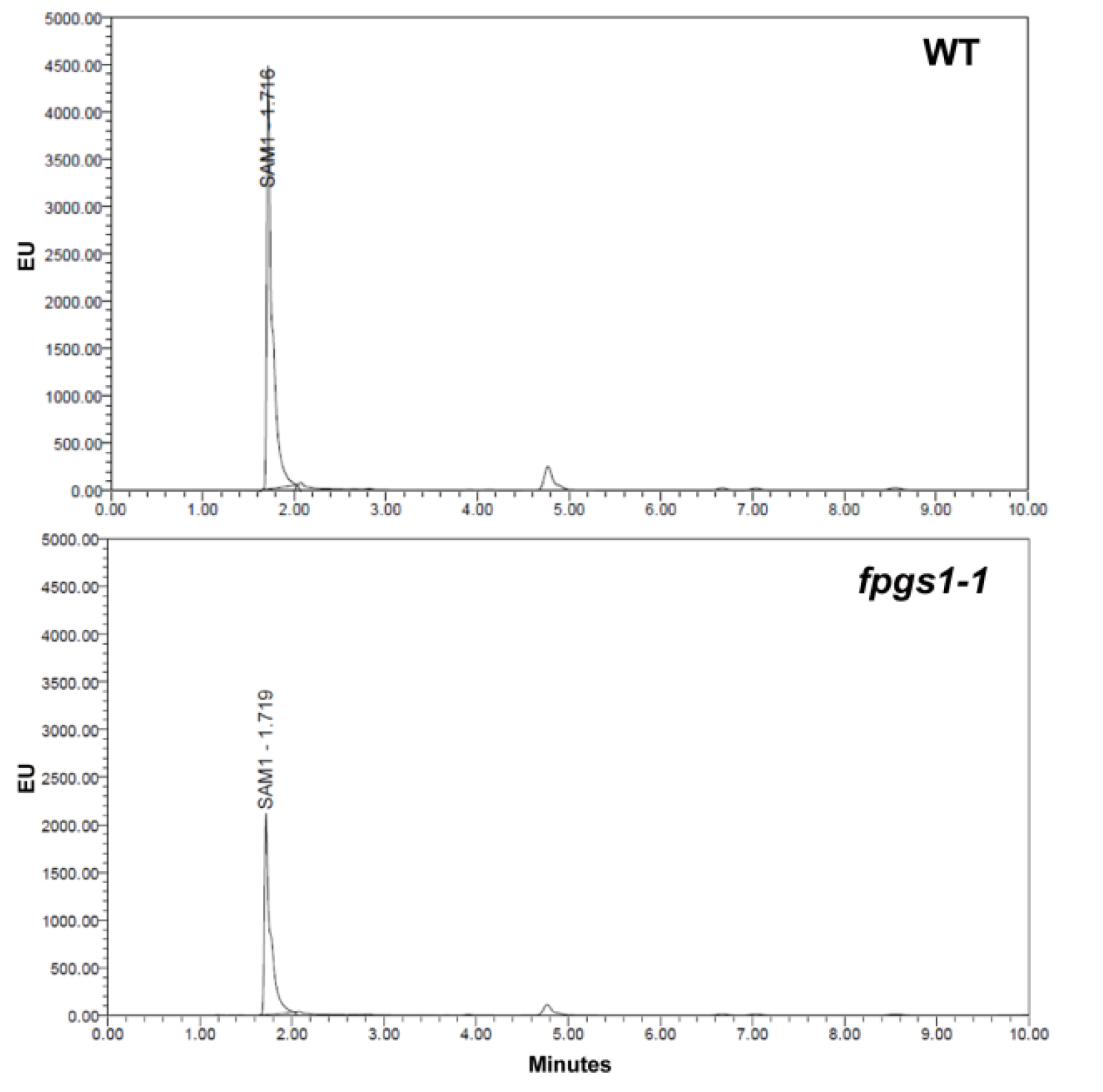

Supplement: Supplementary file 4 — 10.1186/s13068-015-0403-z Chromatographic profiles of wild-type and fpgs1 stem tissue samples analyzed for AdoMet content. Analysis was according to Castro et al. [37]. [file 13068_2015_403_MOESM4_ESM.tif]

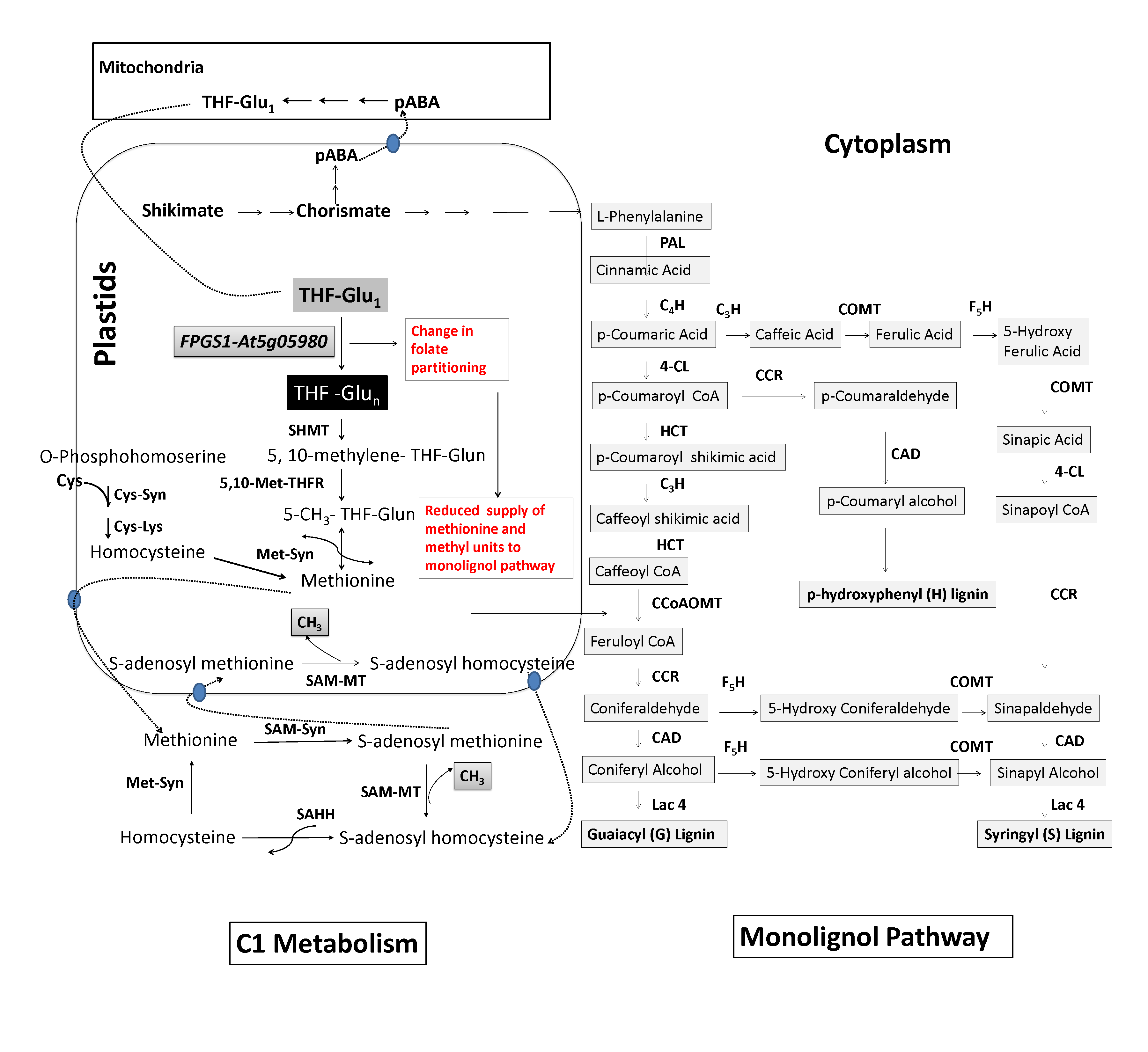

Supplement: Supplementary file 6 — 10.1186/s13068-015-0403-z Hydrothermal pretreatment releases more xylose and glucose from fpgs1 biomass than from wild-type biomass. (A) Glucan and xylan contents of Arabidopsis wild-type and fpgs1-1 stem biomass. (B) Total xylose (monomer plus oligomers) released by cellulase and xylanase (150 mg protein/g structural sugars in biomass) after hydrothermal pretreatment at 180 °C for 11.1 min. (C) Glucose and xylose released by cellulase and xylanase (150 mg protein/g structural sugars in biomass) without pretreatment. Error bars are SE, n = 4. [file 13068_2015_403_MOESM6_ESM.tiff]

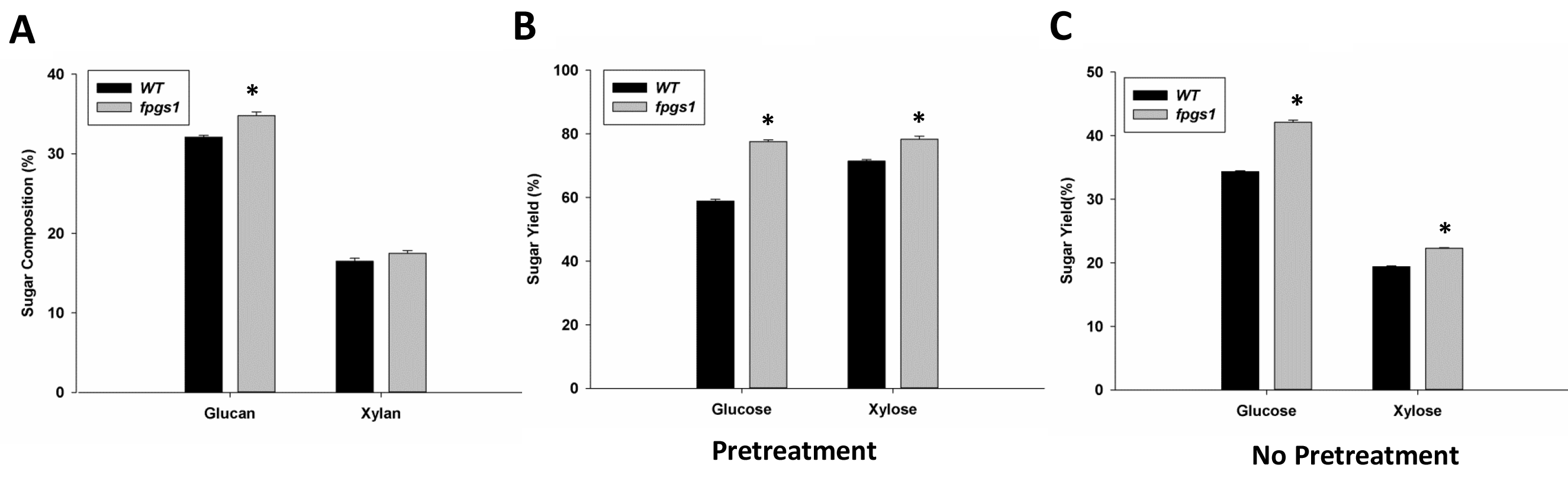

Supplement: Supplementary file 7 — 10.1186/s13068-015-0403-z Comparative aerial growth analysis of fpgs mutants, complemented FPGS1 line (FPGS1c) and wild-type plant. The growth experiment included wild-type plant, two independent mutant lines of fpgs1 (fpgs1-1 and fpgs1-3), knockout mutant of other two isoforms of FPGS genes (fpgs2-mitochondiral and fpgs3-cytoplasmic) and complemented transgenic lines of fpgs1-1 [FPGS1c (pFPGS1::FPGS1-GFP::fpgs1-1)]. Samples were collected at 35 days after germination. Collectively no visual differences were observed in the above-ground growth of these plants (A). No significant differences were observed in per plant fresh weight (B) and dry biomass (C). [file 13068_2015_403_MOESM7_ESM.tif]

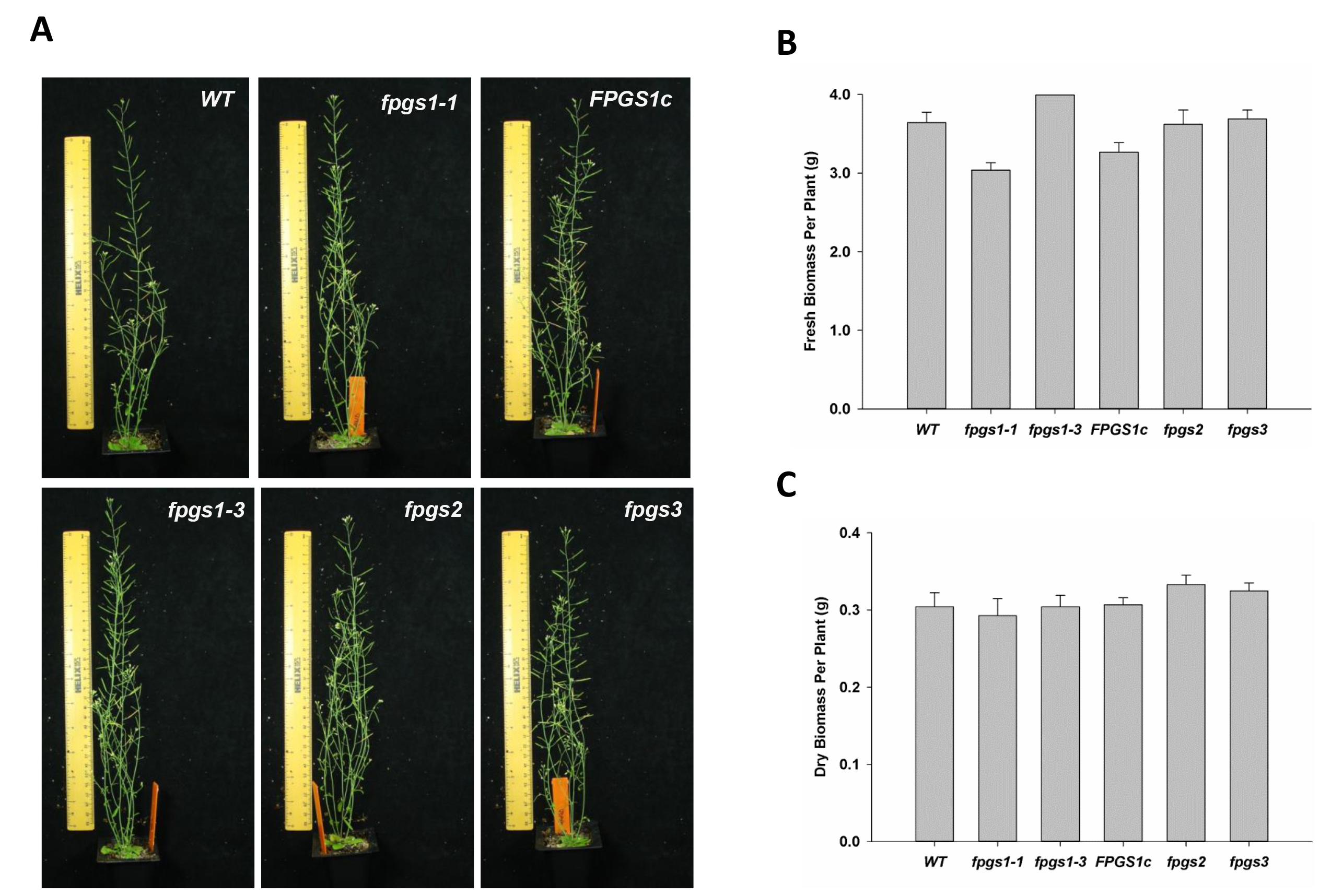

Supplement: Supplementary file 8 — 10.1186/s13068-015-0403-z Schematic representation of C1 metabolism association with the monolignol biosynthetic pathway (Adapted from Humphreys and Chapple [91], Ravanel et al. [56]; van den Broeck et al. [16]. Arrows represent enzymatic reactions and abbreviations depicted next to arrows are the name of the enzyme that catalyzes the associated reaction. (PAL) l-Phenylalanine ammonia lyase; (C4H) Cinnamate 4-hydroxylase; (4-CL) 4-coumarate:coenzyme A ligase; (CCR) Cinnamoyl-CoA reductase; (HCT) Hydroxycinnamoyl CoA: shikimate hydroxycinnamoyltransferase; (C3′H) 4-Coumaroyl-shikimate 3′-hydroxylase; (CCoAOMT) Caffeoyl-CoA-3-O-methyltransferase; (CAD) Cinnamyl alcohol dehydrogenase; (Lac 4) Laccase 4; (F5H) Ferulate 5-hydroxylase; (COMT) Caffeic acid 3-O-methyltransferase; (FPGS1) Folypolyglutamate synthetase 1; (SHMT) Serine hydroxymethyltransferase; (5,10-Met-THF) 5,10-Methylene-tetrahydrofolate reductase; (Met-Syn) Methionine synthase; (SAM-Syn) S-Adenosyl methionine-Synthase; (SAM-MT) S-Adenosyl methionine dependent Methyl transferase; (SAM-HCT) S-adenosyl homocysteinase; (SAHH) S-Adenosyl-L-homocysteine hydrolase; (Cys) Cysteine; (Cys-Syn) Cystathionine synthase; (Cys-Lys) Cystayhioninelyase. [file 13068_2015_403_MOESM8_ESM.tif]
