# Supplementary material for: Loss of function of folylpolyglutamate synthetase 1 reduces lignin content and improves cell wall digestibility in Arabidopsis
Source: Biotechnol Biofuels. 2015 Dec 21;8:224. doi: 10.1186/s13068-015-0403-z (PMC4687376; doi:10.1186/s13068-015-0403-z)
Supplement: Supplementary file 9 — 10.1186/s13068-015-0403-z Primer sequences used for genotyping, plasmid construction, and gene expression analysis by qRT-PCR. [file 13068_2015_403_MOESM9_ESM.pdf]

Table S2. Primer sequences used for genotyping, plasmid construction, and gene expression analysis by qRT-PCR.

Gene Name	Sense (5'-3')/Left prmer	Antisense (5'-3')/Right prmer	Usage
**SALK_015472**	GGCTTTCGAACTGGACTCTTC(LP)	TTAAGGATTTGGGTTCCGATC(RP)	Genotyping
**SAIL_556_GO8**	CTGACCACTTAACAGCCTTGC(LP)	ATTGGAGGTTGGATTAGGTGG(RP)	Genotyping
**SALK008883**	CGATGGTTCTCTTCTGCAGTC(LP)	GGAGGGTTCTCACTTCTCATG(RP)	Genotyping
**SAIL510E12**	GGCCCAAGTTGTTTGTATCATC(LP)	TTCAAGCTTGTCACGATTTCC(RP)	Genotyping
**SAIL151E09**	CTCTTTCTGCGGTTCAGACAC(LP)	TGATCCCTTCAAAACGATCAC(RP)	Genotyping
**pFPGS1**	TGTTAAGGTCAAAACATAAACTCCAT	TCTCTTGTCACTAACATTGCTACACTT	2 Kb - Promoter
**pFPGS-FPGS1**	TGTTAAGGTCAAAACATAAACTCCAT	TTTTCTGATTAATCTCAGTACATCGC	7kb - Promoter-Gen e
**AtFPGS1**	GGAAAGATTTCGTTTGGATGG	TGACATACAATTGAACAACAATATC	Real Time
**AtFPGS2**	GGGGCTTGACCATACACTGA	CTGCAGGTCCACCACATTG	Real Time
**AtFPGS3**	GCAAAGAAGAAGAGAAGAGTTTCG	GAAAACGAACTTGTTTACTTTGGC	Real Time
**EIF4A2**	GGCTGAATGAAGTTCTCGATGGACAG	ACGAGAGCCTGGCACTGGAGAAG	Real Time - Control
**CCoAMT-AT1 G67980**	CAGTACATCATGGAAACGTCAGC	GGTAATACGGCCATCTTCAGG	Real Time
**Laccase4-AT2G38080**	CACTGGCACGGTGTGAGA	CATTCACCTAGAACGATGACTTTCT	Real Time
**COMT-AT1G33030**	CTTCTCCAGGAATTGAGCATG	GTGTCTCCTGGGAACTCTGG	Real Time
**C3H-AT2 G40890**	GTAACCTTCCTGAAAACAGAGCA	GCTATTGACAGTGAAGCACCTAGC	Real Time
**IGMTI-AT1G21100**	GTCTTCCTTAATACCTGGGCAC	CATCAACTAAAACTTTCACACCTTTG	Real Time
**HMT3-AT3 G22740**	GTTACAAAGGTGCACTTGGATTAC	CATGAACCCTTGGTGCATC	Real Time
**M4T3-AT2 G36880**	TAGATCCACTTGCAGGGAGATT	TATCTTCAGGCTTCTTGGTCAAA	Real Time
**SAMS3-AT3G17390**	TCGTAAAACATGCCGTGAGA	CCTCCTCTGGCTTCTTGGT	Real Time
**SAMS2-AT4G01850**	TAATGGCATGGCTCGCAG	GAATGTCTCTTTCACGATCTTCAG	Real Time
**SAM-MT-AT1 G69526**	GATTCGAGCTTCACGAGATCA	GAAATGTTTCTGAAACATCCTTGG	Real Time
**SAM-MT-AT1 G66690**	GTCATCGTATCAGAGAGCTTTGTT	CTCTAAGATACTTCTCTTCCACGGC	Real Time
**SAM-MT-AT1 G55450**	CCATCTTGTTCCAAAACTTGC	GGTGCGAGATCGAAGTAGTGA	Real Time
**SAM-MT-AT3 G54150**	TGGGCTTGTGGAGCATTAC	GTCGAAGAAATGGACAGCTTG	Real Time
**SMT2530-AT4G22530**	CATCGGAATCGCGGAAC	CCACAGAGTTCTCTCCTCCAAT	Real Time

**SAM-MT-AT1G15125**	ACGTCTCCCACACTGATTTC	GTGTAGTGAATCCTGCCTCTG	Real Time
**SAMS1-AT1G02500**	ACCATCTTCCACTTGAACCC	GCGTATGAGACCTGAACAAGAG	Real Time
**CCR1-AT1G15950**	CCGGAACAAATGGTGGAG	CAATACCAATTCTTGGTGTTTTTG	Real Time
**PAL3-AT5G04230**	CCGCTTCAGAAACCTAAACAAG	GCTAGACGAGTGTTATCCATGG	Real Time
**CAD1-AT1G72680**	CCTGGGCATGAGATTGCT	GCAGTACCTTTCATGAACAACAAT	Real Time
