# Supplementary material for: Loss of function of folylpolyglutamate synthetase 1 reduces lignin content and improves cell wall digestibility in Arabidopsis
Source: Biotechnol Biofuels. 2015 Dec 21;8:224. doi: 10.1186/s13068-015-0403-z (PMC4687376; doi:10.1186/s13068-015-0403-z)
Supplement: Supplementary file 10 — 10.1186/s13068-015-0403-z List of cell wall monoclonal antibodies. [file 13068_2015_403_MOESM10_ESM.pdf]

Appendix S1- List of Cell Wall Monoclonal Antibodies Used in Glycome Profiling

Appendix S1: Detailed list of plant cell wall glycan-directed monoclonal antibodies (McAbs) used in this study for ELISA analyses (Figure 6a). The groupings of antibodies are based on a hierarchical clustering analysis of all mAbs screened against a panel of plant polysaccharide preparations (Pattathil et al., 2010) that groups the mAbs according to the polysaccharides that they predominantly recognize. The majority of listings link to the Wall*Mab*DB plant cell wall monoclonal antibody database (http://www.wallmabdb.net) that provides detailed descriptions of each McAb, including epitope structure recognized (where known), supplier information, and any related literature reference

Glycan Group Recognized	mAb Name
Non-Fucosylated Xyloglucan	CCRC-M54
	CCRC-M48
	CCRC-M49
	CCRC-M96
	CCRC-M50
	CCRC-M51
	CCRC-M53
	CCRC-M100
	CCRC-M103
	CCRC-M58
	CCRC-M86
	CCRC-M55
	CCRC-M52
	CCRC-M99
	CCRC-M95
	CCRC-M101
	CCRC-M104
	CCRC-M89
	CCRC-M93
	CCRC-M87
	CCRC-M88
	CCRC-M57
	CCRC-M90
Fucosylated Xyloglucan	CCRC-M102
	CCRC-M39
	CCRC-M106
	CCRC-M84
	CCRC-M1

Appendix S1- List of Cell Wall Monoclonal Antibodies Used in Glycome Profiling

Group	Antibodies
Xylan 1/XG	CCRC-M111<br>CCRC-M108<br>CCRC-M109
Xylan 2	CCRC-M119<br>CCRC-M115<br>CCRC-M110<br>CCRC-M105<br>CCRC-M117<br>CCRC-M113<br>CCRC-M120<br>CCRC-M118<br>CCRC-M116<br>CCRC-M114<br>CCRC-M154<br>CCRC-M150
Xylan 3	CCRC-M160<br>CCRC-M137<br>CCRC-M152<br>CCRC-M149<br>CCRC-M144<br>CCRC-M146<br>CCRC-M145<br>CCRC-M155
Xylan 4	CCRC-M153<br>CCRC-M151<br>CCRC-M148<br>CCRC-M140<br>CCRC-M139<br>CCRC-M138
Galactomannan	CCRC-M75<br>CCRC-M70<br>CCRC-M74
Homogalacturonan Backbone	CCRC-M131<br>CCRC-M38<br>JIM5<br>JIM136

Appendix S1- List of Cell Wall Monoclonal Antibodies Used in Glycome Profiling

Group	Antibodies
	JIM7<br>CCRC-M34
Rhamnogalacturonan I Backbone	CCRC-M69<br>CCRC-M35<br>CCRC-M36<br>CCRC-M14<br>CCRC-M129<br>CCRC-M72
Linseed Mucilage Rhamnogalacturonan I	CCRC-M40
Physcomitrella Pectin	CCRC-M98<br>CCRC-M94
Rhamnogalacturonan IA	CCRC-M5<br>CCRC-M2
Rhamnogalacturonan IB	CCRC-M23<br>CCRC-M17<br>CCRC-M19<br>CCRC-M18<br>CCRC-M56<br>CCRC-M16
Rhamnogalacturonan IC	JIM137<br>JIM101<br>CCRC-M61<br>CCRC-M30
Rhamnogalacturonan I Arabinogalactan	CCRC-M60<br>CCRC-M41<br>CCRC-M80<br>CCRC-M79<br>CCRC-M44<br>CCRC-M33<br>CCRC-M32<br>CCRC-M13<br>CCRC-M42<br>CCRC-M24<br>CCRC-M12

Appendix S1- List of Cell Wall Monoclonal Antibodies Used in Glycome Profiling

	CCRC-M7<br>CCRC-M77<br>CCRC-M25<br>CCRC-M9<br>CCRC-M128<br>CCRC-M126<br>CCRC-M134<br>CCRC-M125<br>CCRC-M123<br>CCRC-M122<br>CCRC-M121<br>CCRC-M112<br>CCRC-M21<br>JIM131<br>CCRC-M22<br>JIM132<br>JIM1<br>CCRC-M15<br>CCRC-M8<br>MH4.3E5<br>JIM16
Arabinogalactan 1	JIM93<br>JIM94<br>JIM11<br>MAC204<br>JIM20
Arabinogalactan 2	JIM14<br>MAC207<br>JIM19<br>JIM12<br>CCRC-M133<br>CCRC-M107
Arabinogalactan 3	JIM4<br>CCRC-M31<br>JIM17<br>CCRC-M26<br>JIM15<br>JIM8<br>CCRC-M85

Appendix S1- List of Cell Wall Monoclonal Antibodies Used in Glycome Profiling

	CCRC-M81<br>MAC266<br>PN 16.4B4
Arabinogalactan 4	JIM133<br>JIM13<br>CCRC-M92<br>CCRC-M91<br>CCRC-M78
Unidentified	MAC265<br>CCRC-M97

References

Pattathil, S., Avci, U., Baldwin, D., Swennes, A.G., McGill, J.A., Popper, Z., Bootten, T., Albert, A., Davis, R.H., Chennareddy, C., Dong, R.H., O'Shea, B., Rossi, R., Leoff, C., Freshour, G., Narra, R., O'Neil, M., York, W.S., and Hahn, M.G. (2010) A comprehensive toolkit of plant cell wall glycan-directed monoclonal antibodies. Plant Physiology 153: 514-525.
